# Comparative Analysis of Fecal Microbiomes From Wild Waterbirds to Poultry, Cattle, Pigs, and Wastewater Treatment Plants for a Microbial Source Tracking Approach

**DOI:** 10.3389/fmicb.2021.697553

**Published:** 2021-07-14

**Authors:** Amine M. Boukerb, Cyril Noël, Emmanuelle Quenot, Bernard Cadiou, Julien Chevé, Laure Quintric, Alexandre Cormier, Luc Dantan, Michèle Gourmelon

**Affiliations:** ^1^IFREMER, RBE-SGMM-LSEM, Laboratoire Santé Environnement Microbiologie, Plouzané, France; ^2^IFREMER – PDG-IRSI-SEBIMER, Plouzané, France; ^3^Bretagne Vivante – SEPNB, Brest, France; ^4^IFREMER, ODE-UL-LERBN, Laboratoire Environnement Ressource Bretagne Nord, Dinard, France

**Keywords:** microbiome, wild waterbird, fecal pollution, microbial source tracking, enteric pathogens, environmental pathogens, NGS, qPCR

## Abstract

Fecal pollution in coastal areas is of a high concern since it affects bathing and shellfish harvesting activities. Wild waterbirds are non-negligible in the overall signal of the detectable pollution. Yet, studies on wild waterbirds’ gut microbiota focus on migratory trajectories and feeding impact on their shape, rare studies address their comparison to other sources and develop quantitative PCR (qPCR)-based Microbial Source Tracking (MST) markers to detect such pollution. Thus, by using 16S rRNA amplicon high-throughput sequencing, the aims of this study were (i) to explore and compare fecal bacterial communities from wild waterbirds (i.e., six families and 15 species, *n* = 275 samples) to that of poultry, cattle, pigs, and influent/effluent of wastewater treatment plants (*n* = 150 samples) and (ii) to develop new MST markers for waterbirds. Significant differences were observed between wild waterbirds and the four other groups. We identified 7,349 Amplicon Sequence Variants (ASVs) from the hypervariable V3–V4 region. Firmicutes and Proteobacteria and, in a lesser extent, Actinobacteria and Bacteroidetes were ubiquitous while Fusobacteria and Epsilonbacteraeota were mainly present in wild waterbirds. The clustering of samples in non-metric multidimensional scaling (NMDS) ordination indicated a by-group clustering shape, with a high diversity within wild waterbirds. In addition, the structure of the bacterial communities was distinct according to bird and/or animal species and families (Adonis *R*^2^ = 0.13, *p* = 10^–4^, Adonis *R*^2^ = 0.11, *p* = 10^–4^, respectively). The Analysis of Composition of Microbiomes (ANCOM) showed that the wild waterbird group differed from the others by the significant presence of sequences from Fusobacteriaceae (*W* = 566) and Enterococcaceae (*W* = 565) families, corresponding to the *Cetobacterium* (*W* = 1427) and *Catellicoccus* (*W* = 1427) genera, respectively. Altogether, our results suggest that some waterbird members present distinct fecal microbiomes allowing the design of qPCR MST markers. For instance, a swan- and an oystercatcher-associated markers (named Swan_2 and Oyscab, respectively) have been developed. Moreover, bacterial genera harboring potential human pathogens associated to bird droppings were detected in our dataset, including enteric pathogens, i.e., *Arcobacter*, *Clostridium*, *Helicobacter*, and *Campylobacter*, and environmental pathogens, i.e., *Burkholderia* and *Pseudomonas*. Future studies involving other wildlife hosts may improve gut microbiome studies and MST marker development, helping mitigation of yet unknown fecal pollution sources.

## Introduction

Fecal pollution originating from urban areas, agriculture, and wildlife can significantly impair aquatic systems including coastal areas. This pollution can come from multiple sources, i.e., failing wastewater infrastructure and wastes from upstream livestock animals and wildlife, which are mainly routed by runoff following heavy rain events. Several outbreaks of food poisoning through the consumption of contaminated shellfish or surface water have been reported due to such pollution episodes (Potasman et al., 2002; Yoder et al., 2008; European Food Safety Authority (EFSA), and European Centre for Disease Prevention and Control (ECDC), 2019). In addition, this fecal pollution can also cause economic losses from bathing restrictions and closure of shellfish-harvesting areas.

Human microbial pathogens in water include bacterial pathogens represented by two categories, enteric (mainly attributed to fecal pollution) and environmental (autochthonous within aquatic and terrestrial habitats). Testing for major waterborne pathogens would give straightforward interpretation of human health risk (Harwood et al., 2014), but this is impossible since it is time and cost consuming. To avoid this, culture-based methods of fecal indicator bacteria (FIB; *Escherichia coli* and enterococci) are used to assess fecal pollution levels by regulatory agencies to limit exposition to impairment water bodies. Thus, these FIB have been detected in freshwaters, coastal waters, and shellfish as well as a selection of pathogenic bacteria such as *Salmonella* spp. and *Campylobacter* spp. by culture-based methods, or human viruses such as noroviruses by molecular methods (Yoder et al., 2008; Iwamoto et al., 2010; Westrell et al., 2010; Rince et al., 2018). These microorganisms mainly originated from different fecal sources (Pommepuy et al., 2006), while it remains difficult to determine their precise origin, which limits direct and cost-effective remediation efforts. Thus, molecular methods including qPCR Microbial Source Tracking (MST)-based methods are continually developed to identify fecal pollution sources by targeting specific markers.

The recent developments in MST methods helps in discriminating between human and non-human sources of fecal contamination in environmental waters and distinguishing contamination from different animal species and is particularly useful for non-point or multiple sources. This approach consists of investigating the presence of a source-specific target such as microorganisms (e.g., bacteria and viruses) or chemical compounds (e.g., stanols) associated with specific hosts (Boehm et al., 2013; Harwood et al., 2014; Jarde et al., 2018). An MST marker is considered as efficient if it detects its target when the intended host feces are present in the sample (sensitivity) or it does not detect its target in samples that do not contain the host feces (specificity). An MST marker is usually considered sensitive and specific if both these metrics exceed 80% (Boehm et al., 2013). Library-independent MST based on molecular methods targeting bacteria, such as quantification of host-associated Bacteroidales 16S rRNA gene marker by real-time PCR, was found efficient. A high number of qPCR MST markers were validated and already applied on environmental samples (Seurinck et al., 2005; Reischer et al., 2006; Mieszkin et al., 2009; Jarde et al., 2018). These markers target mainly human, pig, cattle, and pet sources while few markers targeting avian sources have yet been described (Reischer et al., 2006; Mieszkin et al., 2009; Ryu et al., 2012; Boehm et al., 2013; Ohad et al., 2016). For these latest sources, we can enumerate two general avian markers (GFD and AV4143), two gull markers targeting the bacterial species *Catellicoccus marimammalium* (Gull2 and Gull4), and poultry (AV43) and poultry litter (LA35) markers (Lu et al., 2008; Green et al., 2012; Ryu et al., 2012; Weidhaas and Lipscomb, 2013; Ohad et al., 2016).

With the rapid and cost-effective development of the Next-Generation Sequencing (NGS) technologies, our understanding of the enteric microbiomes of human, animals, and birds is significantly increasing (Ohad et al., 2016; Unno et al., 2018). Birds are the most diverse group of amniotic vertebrates with more than 10,000 and 20,000 described species and subspecies, respectively^1^ (checked on February 24, 2021). Since most avian microbiome studies have focused on economically important species, such as chicken and turkey, or the impact of diet and behavior on their diversity, little is known on wild waterbirds (Grond et al., 2018; Youngblut et al., 2019). Expanding fecal microbiome data from the range of avian species, including wildlife, will improve our understanding of such microbiome diversity, for example, in relation to migratory or diet habits (Grond et al., 2018). Few studies described bacterial community structure in wild birds, e.g., a selection of four wild waterbird species in Israel (Laviad-Shitrit et al., 2019), Artic-breeding shorebirds (Artic and sub-Artic of North America) (Grond et al., 2019), Canada geese (Lu et al., 2009), shorebirds (red knots) (Grond et al., 2014; Ryu et al., 2014), gulls in United States (Lu et al., 2008; Koskey et al., 2014), or wild geese in China (Wang et al., 2018). Several studies revealed the dominance of Proteobacteria and Bacteroidetes within the avian gastrointestinal (GI) tract, with Firmicutes typically present in any avian fecal sample with different proportions (Grond et al., 2018; Sun et al., 2018). However, samples from captive poultry harbored higher proportions of Firmicutes than those from wild birds (Waite and Taylor, 2015). In Europe, and especially in France, studies on the characterization of such microbiomes are scarce. On the French coastal areas, a high variety of waterbird species including resident and migratory birds is present, with less than 500,000 birds during the breeding season and more than 1 million birds in winter (Issa and Muller, 2015).

From a microbial source tracking view, Ohad et al. (2016) were the latest to develop the AV4143 and AV43 markers targeting avian and chicken sources, respectively, using NGS data. Hence, such data can be used in studying bacterial community transfer from sources of pollution to sinks using the SourceTracker Bayesian approach (Knights et al., 2011). This method has been successfully used in different countries and conditions, helping in the identification of putative fecal sources of contamination (Henry et al., 2016; Brown et al., 2017).

Wild waterbirds are known as reservoirs of enteric bacterial pathogens such as *Campylobacter* spp. and *Salmonella* spp. (Waldenstrom et al., 2007; Chung et al., 2018; Smith et al., 2020). Studying wild waterbird fecal microbiomes could be useful to overview potential bacterial pathogens carried by those hosts. Thus, bacterial genera including potential human pathogens such as *Clostridium sensu stricto 1*, *Fusobacterium*, *Campylobacter*, and *Helicobacter* were identified within bacterial communities from waterbird species in Israel (i.e., great cormorants, little egrets, black-crowned night herons, and black-headed gulls; Laviad-Shitrit et al., 2019).

In the present study, we aimed to explore fecal microbiomes and develop new MST toolbox from a selection of wild waterbirds in France. The objectives of this study were (1) to characterize the fecal bacterial communities of several wild waterbirds within different families such as Anatidae, Laridae, Haematopodidae, Scolopacidae, Phalacrocoracidae, and Hydrobatidae; (2) to compare one fecal microbiome to another from livestock animals (cattle and pigs), poultry, and wastewater samples from wastewater treatment plants (WWTPs); (3) to develop novel bacterial MST qPCR markers targeting specific waterbird species; and (4) to evaluate the presence and distribution of bacterial genera (including enteric and environmental pathogens) within our metabarcoding dataset.

## Materials and Methods

### Fecal Sample Collection and Location

Fresh fecal samples from wild waterbirds, poultry, cattle, pigs, and WWTPs (*n* = 425) were collected from July 2014 to November 2017 from different regions in France, mainly from Brittany: 80.4% of wild waterbirds, 40.3% of poultry, 100% of pigs, 100% of cattle, and 100% of WW. The samples from the other regions were collected from Nouvelle-Aquitaine (18.2% of wild waterbird and 29.8% of poultry samples), Normandy (25.4% of poultry samples), and Occitanie (0.4% of wild waterbird and 4.5% of poultry samples). Wild waterbird samples (*n* = 275) were collected from July 2016 to October 2017, and represented by six families and 15 species, with two genera for which the species was not always specified (i.e., *Larus* spp. and *Chroicocephalus* spp.; Supplementary Tables 1, 2). More precisely, the families of wild waterbirds are as follows: (i) Laridae [86; 83 Larinae (subfamily), seagulls and gulls: 45 *Larus* spp., 15 *Larus argentatus*, 7 *Larus marinus*, 3 *Chroicocephalus* spp., 13 *Chroicocephalus ridibundus*, and 3 Sternidae (subfamily): terns (*Thalasseus sandvicensis*)], (ii) Phalacrocoracidae [24 cormorants; 21 great cormorants (*Phalacrocorax carbo*) and 3 European shags (*Phalacrocorax aristotelis*)], (iii) Scolopacidae [5 dunlins (*Calidris alpina*), 11 red knots (*Calidris canutus*), and 11 curlews (*Numenius arquata*)] and Haematopodidae [23 oystercatchers (*Haematopus ostralegus*)] (these two families are part of wader-type birds), (iv) Hydrobatidae (two storm-petrels; *Hydrobates pelagicus*), and (v) Anatidae [113; 20 mallards (*Anas platyrhynchos*), 56 Brent goose (*Branta bernicla*), 12 wild swans (*Cygnus olor*), and 25 common shelducks (*Tadorna tadorna*)]. More details such as the geographical coordinates of the wild bird sample collection sites and their diets can be obtained from Supplementary Tables 1, 2 and the metadata Supplementary Table 3, respectively.

In addition, this collection included fecal samples from poultry (droppings or litters; *n* = 67) and non-avian sources: livestock animals (33 cattle and 37 pigs; both feces and manure) and 13 (9 input and 4 output) WWTP water samples (Supplementary Table 1).

For wild waterbird samples, we distantly observed them until they defecated and flew away, and the fecal samples were immediately collected. For some species, samples were also collected from chicks in their nest. It should be noted that the overall number of samples for the different avian species is variable due to the difficulty in collecting feces from some wild waterbird species that are randomly distributed within the sampling dates. Poultry litters and cattle and pig manures were sampled in farms or in a research institute (ANSES, Ploufragan, France). Wastewater samples were collected at the input and output of three WWTPs. All samples were collected aseptically; then, they were transported to the laboratory on dry ice within 24 h of collection. The samples collected outside the Brittany region were sent to the laboratory on ice within 2 days. All samples were aseptically homogenized. For solid samples, aliquots of about 0.25 g wet weight (droppings, litters, slurries, or manures) were stored at −80°C prior to total DNA extraction. For liquid samples, 10 ml (6 out of the 24 pig slurry samples), 20 or 50 ml (influents of WWTP), and 50 or 100 ml (effluents of WWTP) were filtered onto 0.45-μm nitrocellulose filters. For the 18 additional pig slurry samples, 45 ml was centrifuged at 8,000 rpm for 15 min and supernatants were discarded. Filters and pellets were also stored at −80°C prior to total DNA extraction.

### Total DNA Extraction

Microbial genomic DNA was extracted directly from about 0.25 g of fecal material for solid samples and from filters or pellets for liquid samples using the FastDNA^TM^ Spin Kit for Soil (MP Biomedicals, Illkirsh, France) according to the manufacturer’s recommendations. DNA was eluted in a final volume of 100 μl of sterile DNA/RNA-free water. The quality and quantity of DNA were determined using spectrophotometry (NanoDrop) and a Qubit fluorometric system (Thermo Fisher Scientific). All extracts were stored at −80°C prior to the 16S rRNA amplicon library preparation and sequencing.

### 16S rRNA Library Generation and MiSeq Sequencing

Amplification of the V3–V4 hypervariable region of the 16S rRNA loci was performed using the primer set PCR1F_460 (5′-ACGGRAGGCAGCAG-3′) and PCR1R_460 (5′-TACCAGGGTATCTAATCCT-3′) (Andersson et al., 2008; Liu et al., 2008). The 50-μl final-volume PCR1 reactions contained 5 × PCR buffer (Phusion), 10 mM of dNTP, 0.5 μM of each primer, 5 U of Taq Phusion, and 6 μl of genomic DNA. PCR conditions were as follows: one predenaturation step at 95°C for 5 min, 30 cycles of denaturation at 95°C for 20 s, annealing at 65°C for 30 s, and extension at 72°C for 30 s, one post-elongation step at 72°C for 5 min, and then 4°C forever. PCR product quality and integrity were determined using 1% agarose gel electrophoresis. PCR product purification and secondary PCR amplification for the addition of the Illumina compatible sequencing adapters and unique per-sample indexes were conducted at GenoToul facility (Toulouse, France). Barcoded amplicons were quantified, quality-checked, normalized, pooled, and sequenced within two sequencing runs (May and November 2017) using the 2 × 250 paired-end method on an Illumina MiSeq instrument with a MiSeq Reagent Kit V3 chemistry (Illumina), according to the manufacturer’s recommendations.

### Bioinformatics

#### Bacterial Community Analysis

Raw data were analyzed using the SAMBA v2.0.0 workflow^2^, a Standardized and Automatized MetaBarcoding Analysis workflow using DADA2 (Callahan et al., 2016) and QIIME2 (Bolyen et al., 2019) with default parameters unless otherwise indicated. This workflow developed by the SeBiMER (Ifremer’s Bioinformatics Core Facility) is an open-source modular workflow to process eDNA metabarcoding data. SAMBA was developed using the NextFlow workflow manager (Di Tommaso et al., 2017) and built around three main parts: data integrity checking, bioinformatics processes, and statistical analyses. Firstly, a SAMBA checking process allows one to verify the raw data integrity. Afterward, sequencing primers were trimmed from reads, and reads where primers were not found have been removed. Then, DADA2 was used to filter bad quality reads, correct sequencing errors, overlap paired reads, infer Amplicon Sequence Variants (ASVs), and remove chimeras. Due to the known diversity overestimation generated by DADA2, an additional step of ASV clustering [Operational Taxonomic Unit (OTU) calling] has been performed using dbOTU3 algorithm (Olesen et al., 2017). Taxonomy classification was achieved using the SILVA database 132 (Quast et al., 2013; Glockner et al., 2017). Finally, SAMBA performs extensive analyses of the alpha- and beta-diversities using homemade R scripts (R Core Team, 2020). ASV abundances for each sample were generated at the phylum, family, and genera taxonomic levels. Bacterial community indices describing the alpha diversity included Chao1 and Shannon indices. Beta diversity analyses were achieved by ordination method using non-metric multidimensional scaling (NMDS) with Bray–Curtis and weighted UniFrac distance matrices (Lozupone and Knight, 2005). Significant differences in variance for each index depending on the group/species were tested by Kruskal–Wallis rank sum test and Dunn’s *post hoc* test. Differences in microbial mean taxa abundance according to group/species were detected using ANCOM (Analysis of Composition of Microbiomes), with W value corresponding to the number of times an ASV abundance is significantly different for a group of samples (Mandal et al., 2015). Unique and overlapping ASVs to sample groups were plotted with UpsetR v.1.4.0 (Conway et al., 2017) by highlighting the associated taxonomy in each set.

#### Identification of Potential New MST Markers Based on NGS Data

In order to develop new MST qPCR markers, we investigated unique and host-associated ASVs in a group of samples belonging to a bird species, a bird family, or to a main group of samples (i.e., waterbirds, poultry, cattle, pigs, or wastewaters) using ANCOM. A validation of the retrieved specific/host-associated sequences from non-targeted sources was achieved by building a phylogenetic tree using sequences from already validated markers retrieved from GenBank (i.e., Pig2Bac, Rum2Bac, and HF183).

The selected sequences were compared to the NCBI nucleotide database^3^ to retrieve the 20 best hits with the highest host diversity. These sequences were aligned with ClustalX (v2.1) to determine the variable regions (specific to the target sequence) and constant regions (common to non-target sequences). When regions specific to the targeted host were identified, primers and probes were drawn manually or using Primer3 (v4.1.0^4^), OligoCalc (v3.27) (Kibbe, 2007), and Multiple Primer Analyzer (Thermo Fisher Scientific). Then, the sensitivity and specificity of the designed primers and probes were assayed on target and non-target fecal samples using qPCR assays.

#### qPCR Assays

We assayed three potential targets: one ASV belonging to the genus *Romboutsia* and found associated with swans, one belonging to the genus *Bacteroides* associated with oystercatchers, and one belonging to the species *Paeniclostridium sordellii* and associated with cormorants. Two of these proposed candidates did not allow primers drawing due to the absence of regions specific to the target hosts (Anatidae and cormorants). For the ASV sequence belonging to the genus *Romboutsia* (swans), three pairs of primers were designed. For the ASV sequence belonging to the genus *Bacteroides* (oystercatchers), a pair of primers and a probe have been designed.

Details on the marker genes, primer/probe sequences, and qPCR reaction conditions are listed in Table 1. For the Swan_2 marker, quantitative PCR assays were performed using TaqMan Mix (Invitrogen) with the following conditions: 1 step at 95°C for 10 min followed by 40 cycles of 95°C for 15 s and 60°C for 60 s. Reactions were carried out in a final volume of 25 μl with a final concentration of 300 nM of each primer and 200 nM of probe (Eurogentec, France) and 2 μl of DNA template. DNA samples were tested at 1/10 and 1/100 dilutions, and the appropriate dilution (weaker dilution without inhibition) was retained. Negative controls (no template DNA) were performed in triplicates for each run. A targeted synthetic oligonucleotide, gBlocks Gene fragment (IDT, Integrated DNA Technology), containing a 400-bp partial sequence of 16S rRNA gene of *Romboutsia* was used as standard at 10-fold dilutions ranging from 10^5^ to 10 copies/qPCR. Correlation coefficients (*r*^2^) for all the standard curves were > 0.99 and PCR efficiency ranged between 93.7% and 101.6%. Inhibition tests were performed by running serially diluted DNA templates (10- and 100-fold dilution).

**TABLE 1:** qPCR target genes, primer/probe sequences, and reaction conditions for MST candidates Swan_1, Swan_2, and Swan_3 targeting swans, and Oyscab targeting oystercatchers.

Marker	Primers/probe	Sequences (5’-3’)	Product size (bp)	Reaction conditions	Sensitivity (*n* of samples)/specificity (*n* of samples)
**Swan_2**	Swan_2F	GTAATACGTAGGGGGCAAG	135	1 cycle of 10 min at 95°C and 40 cycles of 15 s at 95°C and 1 min at 60°C	Sensitivity of 75% (*n* = 16 swan feces) and a specificity of 90.2% (*n* = 116 samples)
	Swan_2R	TCTCCTGTACTCAAGTTTAAC			
	Swan_2P	(FAM)-TACGCATTTCACCGCTACAC-(TAMRA)			
**Swan_1**	Swan_1F	GCGGTTTAACAAGTCAGGAG	73	na	Sensitivity 50% (*n* = 6 target samples); specificity 40% (*n* = 5 non-target samples)
	Swan_1R	TACTCAAGTTTAACAGTTTCAAAA			
**Swan_3**	Swan_3F	GGCGGTTTAACAAGTCAGGA	118	na	Sensitivity 83% (*n* = 6 target samples); specificity 0% (*n* = 5 non-target samples)
	Swan_3R	TACGCATTTCACCGCTACAC			
	Swan_3P	(FAM)-ATAGTAAGCTTTTGAAACTGTTAA-(TAMRA)			
**Oyscab**	OYSCAB_F	AAACTCTACGTGTAGGGTCT	206	na	Sensitivity 71% (*n* = 24 target samples); specificity 91% (*n* = 22 non-target samples
	OYSCAB_R	TCAACCGCACTCAAGTACG			

*na, not applicable; F, forward; R, reverse; P, probe.*

#### Identification of Pathogen Groups and a Selection of MST Markers Based on NGS Data

The presence of a selection of 37 bacterial genera known to include enteric or environmental pathogens plus three genera known to harbor validated MST markers were investigated in the whole sequencing dataset. The list for pathogens was validated according to the Canadian ePATHogen risk group database^5^ (accessed on February 17, 2021, Supplementary Table 4).

Then, we investigated co-occurrences of pathogens and a selection of three genera known to harbor MST markers. Relationships among the considered variables were tested using Spearman’s coefficient in R version 4.0.3 (R Core Team, 2020) with statistical significance set at *p* < 0.05, 0.01, and 0.001.

### Data Availability

GenBank accession numbers (BioProject: PRJNA722421) of the 16 ASVs selected as potential MST markers are listed on Supplementary Table 5.

The 16S rRNA dataset generated for this study can be found in the Sequence Read Archive from NCBI (BioProject: PRJNA722421).

Raw data are available on Dataref at Ifremer^6^.

## Results

The identification of bacterial DNA sequences associated with an avian host and used for the development of MST qPCR markers included the acquisition of an original dataset from high-throughput sequencing of 16S rRNA amplicon from avian droppings, livestock animal feces and manure, and WWTP samples. A particular focus was made on wild waterbirds for which less published data are available. More precisely, we collected 275 wild waterbird droppings from 15 different species and five bird families from coastal areas in France, 67 poultry dropping and litter samples, 70 livestock feces and manure (cattle and pigs), and 13 influents/effluents of wastewater samples from WWTPs. The number of samples per species varied from 1 to 56 (mean = 10; Supplementary Table 1).

### Raw Data Primary Analysis

Illumina sequencing of the V3–V4 hypervariable region of the 16S rRNA loci resulted in a total of 17,083,374 reads. After quality checking (deleting low-quality sequences and primers, assembling, and removing chimeras) and ASV clustering, 6,698,670 high-quality reads (31.06%) with an average of 15,761 ± 8,115 reads per sample were retained for downstream analyses. The rarefaction curves of observed ASVs showed that sequencing depth was sufficient to encompass bacterial species richness (Supplementary Figure 1). A total of 7,349 ASVs across the 425 fecal samples have been obtained, where 7,327 ASVs were assigned at the phylum level, 6,999 at the family level, and 6,255 at the genus level.

The main part of the obtained reads originated from wild waterbird fecal samples (*n* = 275) collected from coastal areas [57.1% of the total reads; Anatidae (*n* = 113), 24.4%; Laridae (*n* = 86), 19.7%; Haematopodidae and Scolopacidae (wader birds; *n* = 50), 7.8%; Phalacrocoracidae (cormorants; *n* = 24), 4.5%; and Hydrobatidae (storm petrels; *n* = 2), 0.6%]. The remaining reads were distributed among poultry [*n* = 67; 15.6% of the total reads; domestic Anatidae (ducks, geese, and swans), 5.7%; chickens, 5.5%; turkeys, 2.2%; and guinea fowls, 2.1%], livestock animals (*n* = 70; 22.2% of the total reads; cattle, 8.5%; and pigs, 13.7%), and influents/effluents of wastewater samples (*n* = 13; 5.1% of the total reads).

**FIGURE 1:**
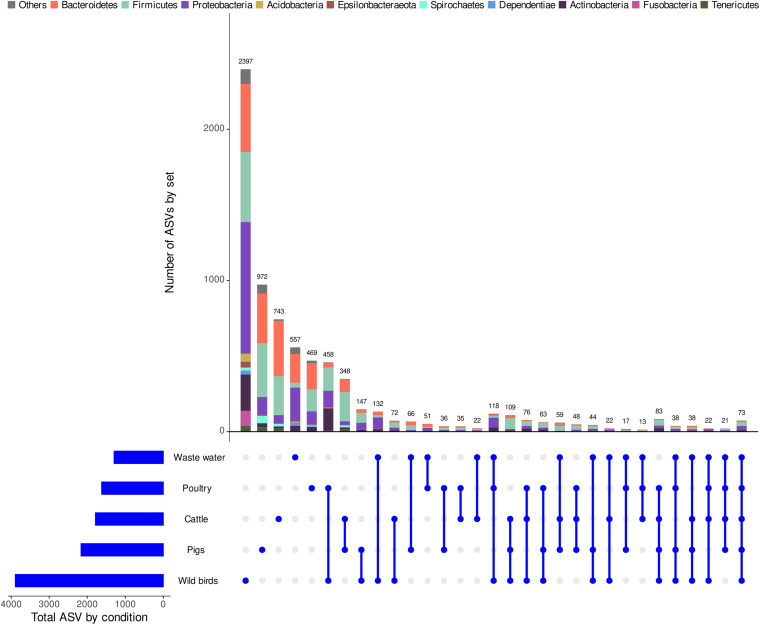
UpSetR visualization of interactions between the obtained ASVs within the whole dataset. The grid along the bottom is used to identify interaction sets (analogous to a Venn diagram). Heavily colored and connected blue dots in the grid indicates that the key group shown on the left has contributed to the interaction set shown on the top. The number of ASVs per group and the size and taxonomy (at the phylum level) of each interaction set are represented by horizontal bars on the left and vertical bars on the figure above, respectively.

Out of the 7,349 ASVs, 69.9% (5,138 ASVs) were specific to one of the five group samples (that is, 32.6% from wild waterbirds, 6.4% from poultry, 10.1% from cattle samples, 13.2% from pig samples, and 7.6% from wastewater samples) (Figure 1). A total of 3,892 ASVs were obtained in wild waterbirds whereas a lowest number of ASVs was obtained in both breeding animals (i.e., 2,162 ASVs and 1,784 ASVs in pig and cattle samples, respectively), in poultry (1,621 ASVs), and in wastewater (1,293 ASVs) samples. Figure 1 shows ASVs shared between two, three, four, or all the five groups. Unique points indicate the signature of a specific ASV for the corresponding group.

### Overall Taxonomic Composition

**FIGURE 2:**
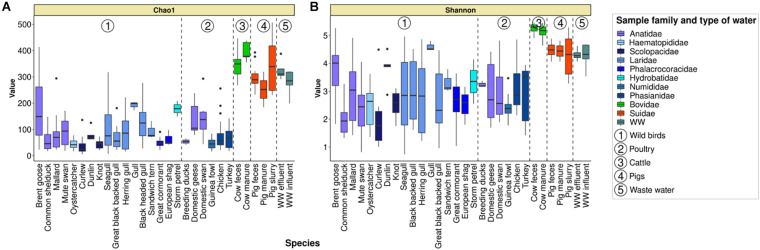
Boxplots illustrating the alpha diversities with variations intra- and inter-groups for Chao1 richness estimator **(A)** and Shannon diversity index **(B)** computed from the ASV contingency table. WW, wastewater.

Two indices were calculated in order to investigate the alpha diversity within our dataset: Chao1 richness estimator (qualitative species richness) and Shannon index (non-parametric quantitative species richness/evenness) (Figure 2). According to the two indices, a low alpha diversity has been observed in both wild waterbird and poultry samples compared to cattle, pigs, and wastewater samples (Kruskal–Wallis rank sum test with Bonferroni’s *post hoc* test; *p* < 0.001; Figure 2): e.g., Chao1 from cattle fecal samples ranged from 268 to 437 ASVs (median value of 358.4), and Shannon ranged from 4.9 to 5.5 (median value of 5.3), while in wild waterbird samples, Chao1 ranged from 5 to 414 ASVs (median value of 52) and Shannon ranged from 0.7 to 5.3 (median value of 2.3).

Overall, bacterial communities from all fecal samples analyzed in this study are composed of 31 different phyla (Supplementary Table 6), with 2 phyla with less than 10 reads, 11 phyla with 10–100 reads, 4 phyla with 100–1,000 reads, 5 phyla with 1,000–10,000 reads, and 9 phyla with >10,000 reads (i.e., Tenericutes, Cloacimonetes, Spirochaetes, Epsilonbacteraeota, Actinobacteria, Bacteroidetes, Fusobacteria, Proteobacteria, and Firmicutes). The six major phyla were represented by Firmicutes (51.8% of the total reads), Proteobacteria (16.6%), Fusobacteria (11.5%), Bacteroidetes (9.1%), Actinobacteria (8.2%), and Epsilonbacteraeota (1.31%).

At the bacterial phyla level, Firmicutes and Proteobacteria and, in a lesser extent, Actinobacteria and Bacteroidetes were present in all the five groups of samples, while Fusobacteria and Epsilonbacteraeota were mainly present in wild waterbirds (98.0% of the total reads of this first phylum; 59.5% in wild Anatidae and 24.3% in Laridae and 78.2% of the total reads of this second phylum; 63.6% in wild Anatidae; Supplementary Figure 2). Nevertheless, Proteobacteria was less represented in wastewater samples, while Bacteroidetes was ubiquitous within non-avian sources: breeding animals [pigs (34.0% of the total reads of this phylum) and cattle (25.1%)] and wild Anatidae (17.0%). Interestingly, Fusobacteria was mostly represented in wild waterbirds.

All other phyla were represented by less than 1% of the total reads. Among these rare phyla, we noticed that the phylum Acidobacteria (3,865 reads) was mainly observed in wild Anatidae (64.1% of the reads of this phylum) and in Laridae (17.5%), and the phylum Cyanobacteria (5,205 reads) was mainly present in Brent geese feces (Anatidae; 83.6%) and, to a lesser extent, in effluents of WWTPs (9.6%). The two phyla Cloacimonetes (16,970 reads) and Fibrobacteres (6,494 reads) were found mainly in pigs (99.7 and 84.8%, respectively), Spirochaetes (22,339 reads) was mainly distributed between pig and cattle samples (77.4 and 20.5%, respectively), and Deferribacteres (*n* = 7,255) was mainly found in wader birds (67%).

**FIGURE 3:**
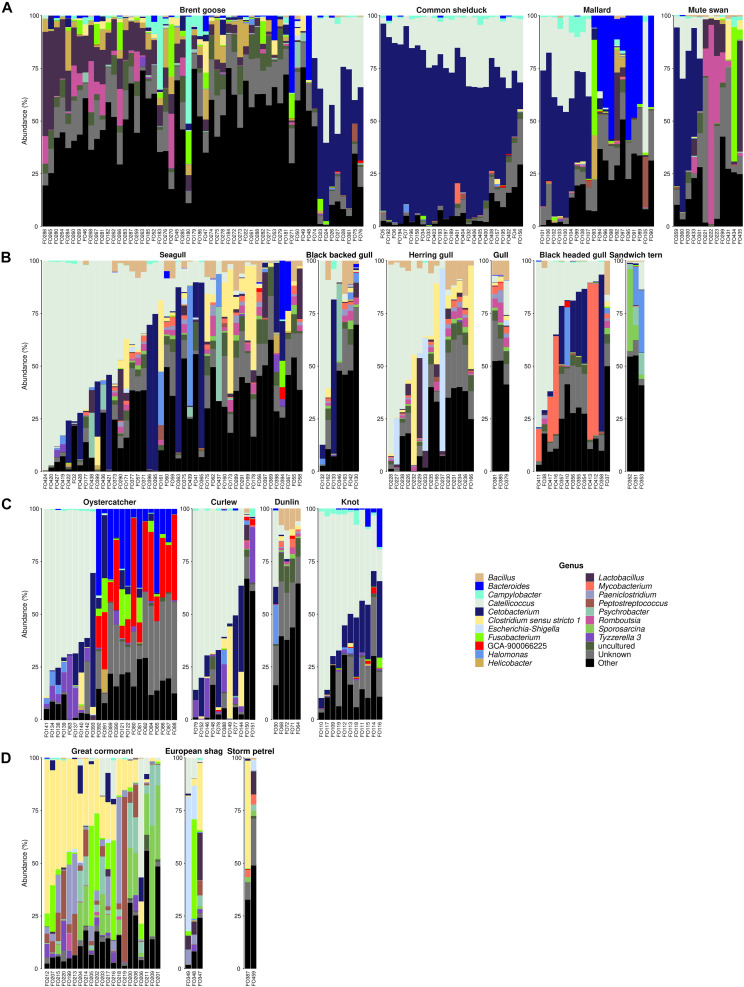
Distribution of predominant bacterial genera in wild waterbird samples according to relative abundance obtained by the gene encoding 16S rRNA. Bacterial community compositions were grouped by wild waterbird families: **(A)** wild Anatidae, **(B)** Laridae, **(C)** Haematopodidae and Scolopacidae (wader birds), and **(D)** Phalacrocoracidae and Hydrobatidae (cormorants and storm petrels, respectively). Stacked bar plots represent the sequence abundances of the 17 most abundant genus-level taxa identified in the fecal samples. Percent sequence abundances given as the number of reads matching a given bacterial family per total reads for that sample.

At the genera level, *Catellicoccus* (Firmicutes; 19.2%) and *Cetobacterium* (Fusobacteria; 17.5%) were the most prevalent genera within wild waterbirds (Figure 3). In poultry, the two dominant genera were *Lactobacillus* (Firmicutes; 26.4%) and *Romboutsia* (Firmicutes; 12.7%; Supplementary Figure 4). In cattle, *Ruminococcaceae* UCG-005 and *Ruminococcaceae* UCG-010 (Firmicutes) were the dominant genera in 61.5 and 38.5% of cattle feces, respectively, whereas *Acinetobacter* was the dominant genus in 71.4% of cattle manures (Supplementary Figure 4B). The main genera for pig feces were *Lactobacillus* (12.6%) and *Clostridium sensu stricto* 1 (Firmicutes; 11.1%), and for pig slurry and solid manure, *Clostridium sensu stricto 1* (25.7 and 7.7%, respectively). Finally, the main genera for influents were C39 (Proteobacteria; 6.4%) and *Acinetobacter* (Proteobacteria; 6.2%), and for effluents, *Arcobacter* (Proteobacteria; 8.7%) and *Mycobacterium* (Actinobacteria; 6.6%; Supplementary Figure 4).

### Comparative Analyses

#### Fecal Microbiome Composition of the Wild Waterbirds

##### Alpha diversity

Significant differences were observed where the highest bacterial community richness (Chao1) among the wild waterbird fecal samples was observed in gulls (*Chroicocephalus* spp.; Laridae; *n* = 3; median value of 199 ASVs), storm petrels (*n* = 2; mean value of 179 ASVs), and Brent geese (Anatidae; *n* = 56; median value of 149) (Figure 2A). The Chao1 index in Brent geese was significantly higher than the ones observed from wader birds such as curlews (*n* = 11) and oystercatchers (*n* = 23) (Scolopacidae and Haematopodidae, respectively), and from common shelducks (Anatidae) (e.g., median value of 24 ASVs in samples from curlews), which both present the lowest bacterial community richness (Kruskal–Wallis rank sum test with Bonferroni’s *post hoc* test; *p* < 0.001). Furthermore, the largest range in values was observed for Brent geese (23–414 ASVs), followed by seagulls (*Larus* spp.; 7–318 ASVs).

The bacterial diversity at the ASV level with Shannon index (Figure 2B), which considers taxon diversity and abundance, also showed significant higher values for samples from Brent geese (median of 4.0) than for curlews (1.8) and common shelducks (1.9; Kruskal–Wallis rank sum test with Bonferroni’s *post hoc* test; *p* < 0.01 and *p* < 0.001, respectively). A high range in Shannon index values (from 0.7, which corresponds to a gull fecal sample almost exclusively composed of the genus *Catellicoccus*, FO424, to 5.0) was observed in seagulls and black-headed gulls.

##### Taxon abundance

**FIGURE 4:**
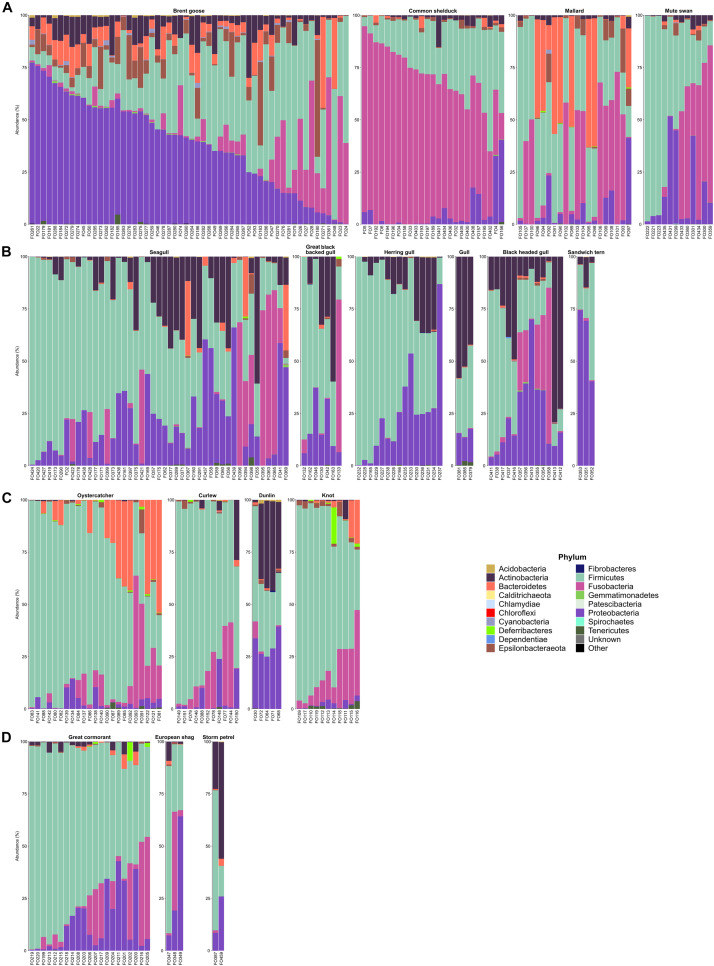
Distribution of the 18 most predominant bacterial phyla in wild waterbird samples according to relative abundances obtained by the gene encoding 16S rRNA. Bacterial community compositions were grouped by wild waterbird families: **(A)** wild Anatidae, **(B)** Laridae, **(C)** Haematopodidae and Scolopacidae (wader birds), and **(D)** Phalacrocoracidae and Hydrobatidae (cormorants and storm petrels, respectively). Stacked bar plots represent the sequence abundances of the 18 most abundant phylum-level taxa identified in the fecal samples. Percent sequence abundances given as the number of reads matching a given bacterial family per total reads for that sample.

The six dominant phyla in wild waterbird fecal samples (*n* = 275) were Firmicutes (45.3% of the total reads of these birds), Proteobacteria (20.1%), Fusobacteria (19.7%), Actinobacteria (7.3%), Bacteroidetes (4.8%), and Epsilonbacteraeota (2.2%; Figure 4 and Supplementary Figure 2). Indeed, Firmicutes was the most abundant phylum in Anatidae (32.0%), Laridae (47.5%), shorebirds (i.e., Haematopodidae and Scolopacidae; 67.7%), and cormorants (Phalacrocoracidae; 69.3%). The second dominant phylum was Fusobacteria in Anatidae and shorebirds (28.0 and 13.8%, respectively), while Proteobacteria predominated in Laridae and cormorants (22.2 and 15.5%, respectively). The third phylum was Fusobacteria in Laridae and cormorants (14.1 and 11.8%, respectively). The phylum Actinobacteria was present in the two storm petrel samples (Hydrobatidae) and Laridae (39.4 and 13.5%, respectively).

Within wild Anatidae, differences were observed according to the bird species with the main presence of Proteobacteria in the Brent geese (53.6% of the samples), while Fusobacteria predominated in common shelducks (88%), mallards (45%), and mute swans (41.7%). Bacteroidetes was the dominant phylum in 30% of mallard samples and Firmicutes in 32.1% of Brent geese, 20% of mallard, and 41.7% of mute swan samples (Supplementary Figure 2A).

Within Laridae, the dominant phylum was Firmicutes in 68.9% of seagulls (i.e., *Larus* spp.), 71.4% of great black-backed gulls, and 86.7% of herring gulls (Supplementary Figure 2B). Actinobacteria predominated in all of the gull’s samples (i.e., *Chroicocephalus* spp.) and 30.8% of black-headed gulls (*Chroicocephalus ridibundus*). Finally, Proteobacteria phylum was predominant in 11.1% of seagulls, 38.5% of black-headed gulls, and 66.7% of sandwich terns.

For wader birds, the dominant phylum was Firmicutes for 78.3% of oystercatchers, all curlews, 20% of dunlins samples, and 90.9% of knots (Supplementary Figure 2C). Bacteroidetes was dominant in 13% of oystercatcher samples while Actinobacteria was dominant in 60% of dunlins.

Concerning cormorants, the dominant phylum was Firmicutes for 90.5% of the analyzed samples and for 33.3% of European shag fecal samples (Supplementary Figure 2D). The two storm petrel samples were slightly different with the predominance of Firmicutes or Actinobacteria.

At the genus level, *Catellicoccus* (Enterococcaceae; 19.2%) and *Cetobacterium* (Fusobacteriaceae; 17.5%) were the most prevalent genera within wild waterbirds (Figure 3), followed by *Clostridium sensu stricto 1* (Clostridiaceae 1; 4.2%), *Lactobacillus* (Lactobacillaceae; 3.9%), *Bacteroides* (Bacteroidaceae; 2.7%), *Fusobacterium* (Fusobacteriaceae; 2.2%), *Romboutsia* (Peptostreptococcaceae; 1.6%), *Mycobacterium* (Mycobacteriaceae; 1.5%), *Psychrobacter* (Moraxellaceae; 1.4%), and uncultured *Ruminococcus* spp., GCA-900066225 (Ruminococaceae; 1.3%).

In wild Anatidae (Figure 3A), *Cetobacterium* was the dominant genus in 8.9% of Brent geese, 92% of common shelducks, 45% of mallards, and 41.7% of mute swans. *Catellicoccus* was dominant in 7.1% of Brent geese, 8% of common shelducks, and 5% of mallard fecal samples. *Bacteroides* was the dominant genus in 40% of mallards and 3.6% of Brent geese, and *Romboutsia* was dominant in 8.3% of mute swans and 5.3% of Brent geese. There is a variable distribution from one sample to another for Brent geese with, e.g., *Lactobacillus* as the dominant genus in 19.6%, *Cetobacterium* in 8.9%, *Catellicoccus* in 7.1%, and *Campylobacter* in 3.6% of the fecal samples, while a quite homogeneous distribution was obtained in the common shelducks with *Cetobacterium* as the most dominant genus followed by *Catellicoccus*.

In Laridae, *Catellicoccus* was the dominant genus in 40% of seagulls (i.e., *Larus* spp.), 28.6% of great black-backed gulls, 40% of herring gulls, and 25% of black-headed gulls (Figure 3B). *Cetobacterium* was the dominant genus in 11.1% of seagulls (i.e., *Larus* spp.), 14.3% of great black-backed gull, and 38.5% of black-headed gulls, while *Clostridium sensu stricto 1* predominated in 11.1% of seagulls and 13.3% of herring gulls. *Mycobacterium* was dominant in 23.1% of black-headed gulls.

Like Laridae, the wader birds group presented the highest values for *Catellicoccus* in 34.8% of oystercatchers, 72.7% of curlews, 20% of dunlins, and 81.8% of knots (Figure 3C). *Cetobacterium* was the second dominant genus in only 0.9% of oystercatchers, 9.1% of curlew, and 20% of knots; *Bacteroides* and GCA-900066225 (uncultured *Ruminococcus* spp. from the Ruminococcaceae family) were dominant in 26.1 and 34.8% of oystercatchers, respectively.

In cormorants, *Clostridium sensu stricto 1* was the dominant genus in 42.8% of great cormorants and 33.3% of European shags, followed by *Sporosarcina* (Planococcaceae), *Fusobacterium*, *Catellicoccus*, and *Paeniclostridium* (Clostridiaceae) that were dominant in 19, 14.3, 4.8, and 4.8% of the fecal samples of great cormorants, respectively (Figure 3D).

#### Fecal Microbiome Composition in Poultry

##### Alpha diversity

The highest bacterial community Chao1 richness among the analyzed fecal samples from poultry was observed within domestic swans (median value of 137.5 ASVs) and domestic geese (105 ASVs), while the lowest bacterial community richness was observed in guinea fowls (45 ASVs) (Figure 2A).

The Shannon index showed the highest values for samples from turkeys (median value of 3.5) and the lowest values for guinea fowls (median value of 2.4) (Figure 2B).

##### Taxon abundance

Poultry samples included two types of samples: dropping samples of hens, turkeys, guinea fowls, and domestic Anatidae, and litter samples (from hens and turkeys). The three dominant phyla in all the samples were Firmicutes (67.9%), Actinobacteria (16.0%), and Proteobacteria (12.8%; Supplementary Figures 2, 3). Among domestic Anatidae, Firmicutes was the dominant phylum for all the three breeding duck samples, for 66.7% of domestic swans, and 66.6% of domestic geese, while Actinobacteria was the dominant phylum for 36.4% of geese and 16.7% of swans. In addition, Firmicutes was the dominant phylum in all guinea fowls, in 73.1% of chicken samples and all the turkeys. Actinobacteria was dominant in 22.2% of the chicken litter samples and Proteobacteria in 29.4% of the chicken dropping samples. Furthermore, Actinobacteria was in higher proportion in poultry litter samples than in droppings (mean percentage value of 4.4% in chicken droppings vs. 30.9% in chicken litter).

At the genus level, the two dominant genera were *Lactobacillus* (26.4%) and *Romboutsia* (12.7%; Supplementary Figure 4). In domestic Anatidae, *Romboutsia* was the dominant genus in 36.4% of geese and 50% of swans, *Turicibacter* (Erisipelotrichaceae) in 27.3% of geese and 33.3% of swans, and *Jeotgalibaca* (Carnobacteriaceae) in 66.7% of breeding ducks. *Lactobacillus* was the dominant genus in all of the 11 guinea fowls, in all of the three turkey feces, in 35.3% of the chicken dropping, and 85.7% of the turkey litter and 11.1% of the chicken litter samples.

A differential bacterial composition between dropping and litter samples was observed. In poultry droppings, the dominant genera were *Lactobacillus* (27.4 and 91.7% in chicken and turkey, respectively), followed by *Pseudomonas* (Pseudomonadaceae), *Acinetobacter* (Moraxellaceae), and *Romboutsia* (at 13.2, 12.6, and 9.4% in chicken, respectively). In chicken and turkey litters, they were represented by *Lactobacillus* (12.8 and 21.2%), *Staphylococcus* (18.2 and 10.0%), *Weissella* (Leuconostocaceae; 14.1 and 6.3%), *Corynebacterium 1* (10.7 and 7.4%), *Brachybacterium* (Dermabacteriaceae; 5.1 and 4.9%), and *Brevibacterium* (Brevibacteriaceae; 7.3 and 2.5%).

#### Fecal Microbiome Composition Within Livestock Samples (Cattle and Pigs)

##### Alpha diversity

As indicated above, the highest alpha diversity according to the two tested indices was observed in cattle fecal samples (see the overall bacterial composition section). These results were observed for both cow feces and cow manure samples (Figure 2).

In pig fecal samples, Chao1 richness showed significant high values than that of cattle samples (Kruskal–Wallis rank sum test with Bonferroni’s *post hoc* test; *p* < 0.001), which was the opposite for Shannon’s index. The greatest bacterial community richness was observed in pig slurry samples (median value of 339 ASVs vs. 289 ASVs in pig feces).

##### Taxon abundance

In breeding animal samples [cattle (*n* = 33) and pigs (*n* = 37)], Firmicutes (62.0 and 65.4%, respectively) and Bacteroidetes (26.9 and 22.5%) were the dominant phyla (Supplementary Figures 2, 3B,C). Among the cattle samples, Firmicutes was the dominant phylum in all the feces and 71.4% of manure samples, while Proteobacteria was dominant within the two other manure samples. Among pig samples, Firmicutes was the dominant phylum for all the feces, all the slurry, and in one pig manure sample, while Bacteroidetes was dominant in the other pig manure samples.

At the genus level, *Ruminococcaceae* UCG-005 and *Ruminococcaceae* UCG-010 were the dominant genera in 61.5% and 38.5% of cattle feces, respectively, whereas *Acinetobacter* was the dominant genus in 71.4% of cattle manures (Supplementary Figure 4B). In pigs, *Clostridium sensu stricto 1* was the main genus in 18.2% of feces, one (50%) manure, and 87.5% of the slurry samples, while *Lactobacillus* was the main dominant genus in 54.5% of feces. DMER64 genus from the Rikenellaceae family was dominant in 12.5% of the slurry samples. *Prevotella 9* and *Escherichia–Shigella* were dominant in two (18.2%) and one (0.9%) feces, respectively, whereas *Acinetobacter* was dominant in the second (50%) pig manure sample (Supplementary Figure 4C).

#### Fecal Microbiome Composition of the Influents and Effluents of WWTPs

##### Alpha diversity

In wastewater samples, Chao1 richness and Shannon index presented significantly higher values than in wild waterbird samples (Kruskal–Wallis rank sum test with Bonferroni’s *post hoc* test; *p* < 0.001; Figure 2). In fact, median values of 296 ASVs for Chao1 estimator and 4.3 for Shannon index were obtained.

##### Taxon abundance

Proteobacteria (38.8%), Firmicutes (22.1%), Actinobacteria (19.2%), and Bacteroidetes (12.4%) were the dominant phyla in wastewater samples (*n* = 13; Supplementary Figures 2, 3D). Proteobacteria was the dominant phylum in all the four effluents and in 55.6% of influents, whereas Firmicutes was dominant in 33.3% of influents and Actinobacteria in one (11.1%) effluent sample.

At the genus level, *Acinetobacter* was the dominant genus in one effluent and three influent samples. *Hypnocyclicus* (Fusobacteriaceae), *Methylotenera* (Methylophilaceae), and *Arcobacter* (Campylobacteriaceae) were the dominant genera in one effluent sample, while C39 (Rhodocyclaceae), *Trichococcus* (Carnobacteriaceae), and *Mycobacterium* (Mycobacteriaceae) were dominant in three, two, and one influent sample, respectively (Supplementary Figure 4D).

### Beta Diversity

#### Within the Overall Dataset

**FIGURE 5:**
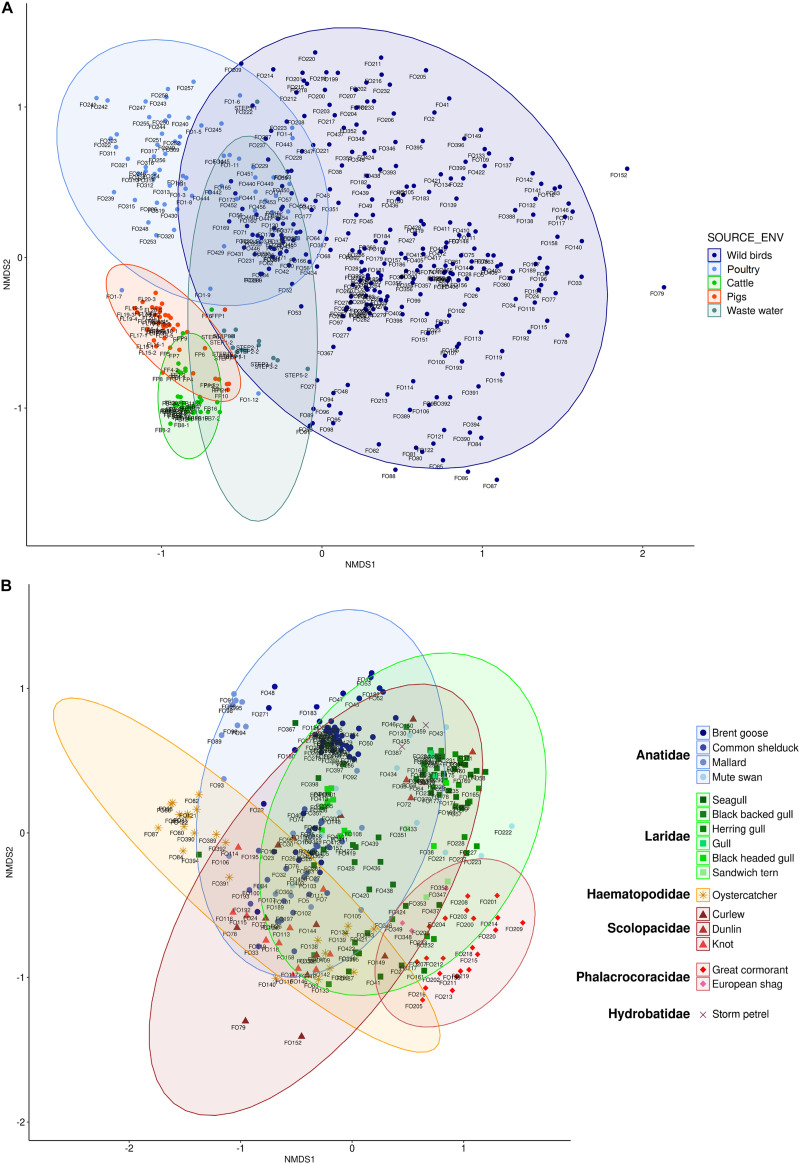
Non-metric multidimensional scaling (NMDS) plots based on Bray–Curtis (**A**, stress = 0.177; **B**, stress = 0.179) distance metrics in relation to the whole dataset **(A)** or the wild waterbird groups **(B)**. Colors represent host classes. Ellipses represent 95% confidence intervals of centroids of each point.

Non-metric multidimensional scaling analysis of the whole dataset using Bray–Curtis distance metric showed that individuals of the same group (i.e., wild waterbirds, poultry, cattle, pigs, or wastewaters) clustered together, with however a greater diversity within wild waterbird samples (stress value 0.177; Adonis *R*^2^ = 0.14; *p* = 10^–4^; Figure 5A). The structure of the bacterial communities was also distinct both according to bird and/or animal species and according to bird and/or animal families (Adonis *R*^2^ = 0.13; *p* = 10^–4^; Adonis *R*^2^ = 0.11; *p* = 10^–4^, respectively). This suggests that each group harbored a distinct bacterial community profile completed by the shared part with the other groups (Figure 5A).

The ANCOM analysis applied to these five groups showed that the wild waterbird group differs mainly from the other groups by the significant presence of sequences from Fusobacteriaceae (*W* = 566) and Enteroccocaceae (*W* = 565) families, more precisely from the *Cetobacterium* (*W* = 1427) and *Catellicoccus* (*W* = 1427) genera, respectively. We noted the low abundance of the Rikenellaceae (*W* = 564) and Christensenellaceae (*W* = 563) families that are mainly detected in pigs and cattle. Poultry can be outlined from the other groups by sequences from Lactobacillaceae (*W* = 566) (mainly from *Lactobacillus*), Dermatobacteriaceae (*W* = 564) (mainly from *Bradybacterium*), and Staphylococcaceae (*W* = 564) (mainly from *Staphylococcus*). Cattle were found characterized by the very low abundance of sequences from Fusobacteriaceae (*W* = 564), Enteroccocaceae (*W* = 563), and Lactobacillaceae (*W* = 561), while Rikenellaceae (*W* = 565) and Christensenellaceae (*W* = 563) were highly present in pigs, with low abundance of sequences from Fusobacteriaceae (*W* = 566). Wastewater samples can be distinguished from the others by the abundance of sequences from Rhodocyclaceae (*W* = 566) and Arcobactericeae (*W* = 562).

#### Within the Wild Waterbird’s Microbiota

Within this group, the samples clustered according to their families (Figure 5B and Supplementary Figure 5). In the same way, the structure of bacterial communities was distinct both according to bird and/or animal species and according to the bird and/or animal families using either Bray–Curtis (stress value 0.179; Adonis *R*^2^ = 0.15; *p* = 10^–4^; Adonis *R*^2^ = 0.14; *p* = 10^–4^, respectively) or weighted-Unifrac distance metrics (stress value 0.126; Adonis *R*^2^ = 0.10; *p* = 0.0035; Adonis *R*^2^ = 0.11; *p* = 0.0005, respectively). For Bray–Curtis distance metric, a high diversity was obtained inside each bird family with, however, a weaker diversity for samples from *Phalacrocoracidae.* For the weighted-Unifrac distance metric, most of the samples clustered together, with a greater diversity for Phalacrocoracidae and Anatidae. Indeed, few samples from geese feces differ from overall other avian samples (Figure 5B and Supplementary Figure 5).

### Identification of Potential New MST Markers From the NGS Data

Following the analysis of the bacterial communities within our dataset, an investigation of the unique and/or host-associated ASVs in a group of samples belonging to the same group or to a bird species was carried out to identify host-associated sequences and thus potentially develop new MST qPCR-based markers.

Among these host-associated ASVs, 17 candidates were retained with four that were further investigated in this study: one ASV belonging to the genus *Romboutsia* and found strongly associated with swans, one belonging to the genus *Bacteroides* and associated with oystercatchers (Supplementary Table 5), one belonging to the species *P. sordellii* and associated with cormorants, and one belonging to the family Peptostreptococcaceae and associated with Anatidae. Two of these proposed candidates did not allow primers drawing due to the absence of regions specific to the target hosts (Anatidae and cormorants). For the ASV sequence belonging to the genus *Romboutsia* (swans), three pairs of primers with the corresponding probes have been designed (Table 1). In addition, a pair of primers has been designed for the ASV sequence belonging to the genus *Bacteroides* (oystercatchers).

The preliminary sensibility and specificity tests carried out for these four pairs of primers on target and non-target fecal samples led us to retain only two pairs, the one targeting swans and named Swan_2, and the one targeting oystercatchers and named Oyscab (Table 1). These markers presented a sensitivity of 75 and 71% and a specificity of 90.2 and 91%, respectively.

### Presence of Potential Pathogenic Bacterial Groups Within the Whole Dataset

**FIGURE 6:**
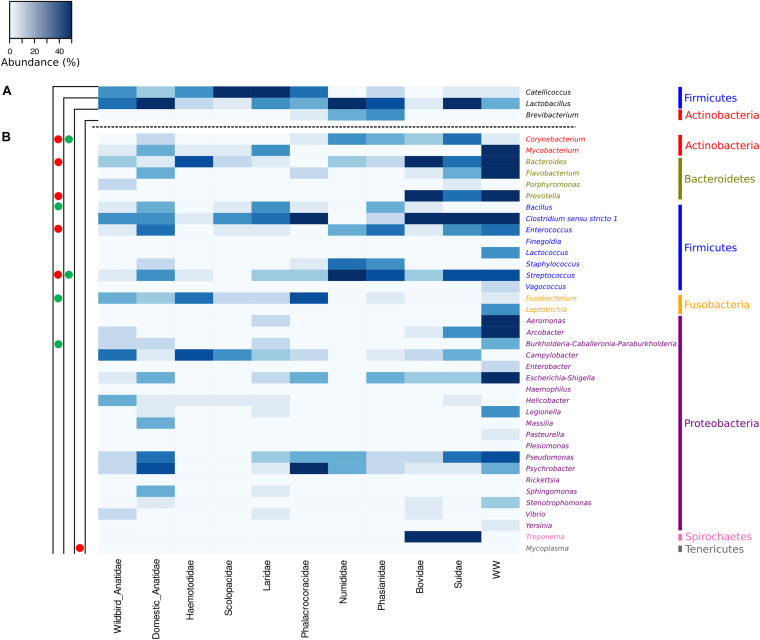
Heatmaps based on the number of reads of a selection of **(A)** 3 genera harboring known MST markers and **(B)** 37 bacterial genera harboring potential pathogens, and derived from the whole dataset at the bird or livestock animal family levels including wastewaters (WW).

We further investigated the distribution of a selection of 37 bacterial genera harboring enteric or environmental human pathogens (Figure 6, Supplementary Figures 6, 7, and Supplementary Table 4). In wild waterbirds (*n* = 275), the members of the genera *Clostridium sensu stricto 1*, *Campylobacter*, *Fusobacterium*, and *Helicobacter* were dominant and present in 67.3, 55.6, 48.7, and 33.8% of the dropping samples, respectively (Supplementary Figure 6B). In poultry, five pathogenic genera were found to be dominant: *Streptococcus*, *Enterococcus*, *Staphylococcus*, *Psychrobacter*, and *Corynebacterium* (79.1, 68.7, 53.7, 50.1, and 49.2%, respectively).

In cattle samples, members of the genus *Bacteroides* were ubiquitous, *Clostridium sensu stricto 1*, *Prevotella*, and *Treponema* were highly prevalent (97%), and *Escherichia–Shigella* in a lesser extent (42.4%). In pigs, *Clostridium sensu stricto 1* and *Treponema* were ubiquitous; *Streptococcus* was present in 81.1% and *Bacteroides* and *Corynebacterium* were present in 75.7% of samples (Supplementary Figure 6B). In wastewater samples, *Bacteroides*, *Arcobacter*, *Flavobacterium*, and *Aeromonas* were ubiquitous, and *Clostridium sensu stricto 1*, *Escherichia-Shigella*, and *Mycobacterium* were present in 92.3% of the 13 samples (Supplementary Figure 6B).

Interestingly, the dominant bacterial genera that include human pathogens were different in wild and domestic Anatidae. In fact, *Campylobacte*r, *Clostridium sensu stricto 1*, *Fusobacterium*, and *Helicobacter* were present in 70.8, 64.6, 54.9, and 54.9% in wild Anatidae, respectively, whereas *Psychrobacter*, *Pseudomonas*, and *Enterococcus* were present in 85, 75, and 70% in domestic Anatidae, respectively. In Laridae, the four dominant genera were *Clostridium sensu stricto 1*, *Bacillus*, *Mycobacterium*, and *Pseudomonas* (75.6, 58.1, 55.8, and 39.5%, respectively). In Phalacrocoracidae, *Clostridium sensu stricto 1* (100%), *Psychrobacter* (91.7%), *Fusobacterium* (83.3%), and *Escherichia–Shigella* and *Pseudomonas* (50%) were dominant. For wader birds and within the Haematopodidae and Scolopacidae families, *Campylobacter* was the dominant bacterial genus that includes human enteric pathogens (78.3 and 63%, respectively), whereas *Bacteroides* (78.3%) and *Fusobacterium* (73.9%) were the most prevalent genera in Haematopodidae and *Clostridium sensu stricto 1* (63%) in Scolopacidae (Figure 6B).

The relationships between the number of reads of ASVs harboring pathogens and ASVs harboring MST markers [i.e., *Catellicoccus* (gull marker), *Lactobacillus* (general avian marker or pig marker, *Lactobacillus amylovorus*), and *Brevibacterium* (poultry litter marker)] were examined by Spearman’s correlation tests.

At the detailed species level, *Brevibacterium* (*p* < 0.05) and *Lactobacillus* (*p* < 0.05) were positively correlated with *Corynebacterium*, while a strong negative correlation for this latest genus was observed with *Catellicoccus* (*p* < 0.001). Only *Catellicoccus* presented negative correlation with three of the four tested genera within Bacteroidetes (*p* < 0.001) (Supplementary Figure 7).

While no correlation was observed for *Lactobacillus* and the tested bacterial genera harboring pathogens, *Brevibacterium* had negative correlations with *Vagococcus* (*p* < 0.05), *Leptotrichia* (*p* < 0.05), and *Pasteurella* (*p* < 0.05) at the five group levels (Supplementary Figure 6). *Catellicoccus* presented negative correlation with *Prevotella* (*p* < 0.05), but positive correlations with *Haemophilus* (*p* < 0.05) and *Mycoplasma* (*p* < 0.05), and strong correlation with *Fusobacterium* (*p* < 0.001).

At the bird or livestock animal family levels, *Lactobacillus* harboring general avian and pig MST markers presented positive correlations with *Corynebacterium* (*p* < 0.05) and *Streptococcus* (*p* < 0.05), while the gull-associated genus *Catellicoccus* had negative correlations with those two genera. In addition, *Catellicoccus* had negative correlations with *Bacteroides* (*p* < 0.05), *Prevotella* (*p* < 0.05), and *Enterococcus* (*p* < 0.05) and positive correlations with *Bacillus* (*p* < 0.01), *Fusobacterium* (*p* < 0.01), and the *Burkholderia–Caballeronia–Paraburkholderia* group (*p* < 0.05) (Figure 6).

## Discussion

Although the 16S rRNA gene amplicon method is a gold standard being used in most microbiome-based studies, only recent works focused on its use in MST (Microbial Source Tracking) development, while its accuracy is questionable in detecting pathogens in routine tests. In this study, we generated an original dataset of highly diverse and predominant wild waterbirds (275/342; 80% of bird fecal samples) with inter-individual replicates for all wild waterbird species investigated (2–56), with different diets (i.e., herbivore, omnivore, and carnivore; Supplementary Table 1) and from different geographical locations in France (at least six different departments). This dataset also considers the other potential sources of fecal bacteria to temperate coastal areas (oceanic climate; Brittany, France) from individual feces of poultry, pigs, and cattle to composite samples under the influence of farm environment (i.e., poultry litter and pig and cattle manure samples) and influents/effluents from WWTPs. In addition, for the Anatidae family, we collected samples from both wild and captive birds.

Wild waterbirds are fascinating animals that present special behavior, dietary patterns, and flight capacities that could influence the diversity of their gut microbiota. This diversity was suggested to be higher than that of mammals, since birds are more diverse in terms of species, with a greater dependence on microbes for digestion and their adaptation to various terrestrial and aquatic environments and to long-distance flights (Hird et al., 2015; Grond et al., 2018). They are an integral part of the aquatic ecosystems, and their fecal pollution should not be neglected since they harbor and spread relevant pathogens and antibiotic resistance determinants (Ewbank et al., 2021). One of the first 16S rRNA gene amplicon studies that attempt to investigate the GI microbiome of wild birds was done by Wienemann et al. (2011), which analyzed seasonal changes in the gut microbial community of wild and captive capercaillie (*Tetrao urogallus*) (Wienemann et al., 2011). A few studies on wild birds followed this publication, where mainly Proteobacteria, Firmicutes, and Actinobacteria were found as the most abundant bacterial phyla (Kreisinger et al., 2015; Laviad-Shitrit et al., 2019; Fu et al., 2020). From an MST view, few studies developed markers for wild waterbird species including ducks (Devane et al., 2007), geese (Hamilton et al., 2006), gulls (Lu et al., 2008), and cranes (Ryu et al., 2012).

Our dataset (>35 different bird or animal species) shows few taxonomic groups to be present in all samples (i.e., Firmicutes, Proteobacteria, Bacteroidetes, and Actinobacteria), in agreement with the dataset on vertebrate microbiome obtained by Youngblut et al. (2019). In addition, this dataset makes available data on avian species that had not been considered in their study (i.e., common shelducks, storm petrels, and European shags). However, the main feature of this dataset is the presence of taxonomic groups that are different between birds, other animals, and wastewaters, and also between wild and captive birds. This differential detection is very useful for developing MST markers targeting specific DNA sequences of a group, family, or species for the purpose of identifying sources of fecal contamination in the environment. It is also noteworthy that in most cases we found high intra-species diversity, suggesting the need to sample enough individuals within the same species. This is particularly relevant for Brent goose and seagulls, whereas individual differences were much lower for common shelducks. Interestingly, our data suggest a lower diversity of the fecal microbiota of wild waterbird compared to that of farm animals and humans (reflected by WW analysis). Dietary and environmental conditions are different between captive and wild animals and might impact this microbiome diversity (Hird, 2017). In addition, species physiology may play a major role with birds presenting a smaller (in terms of surface) and less evolved GI tractus compared to that of farm animals and humans (Reese and Dunn, 2018). Furthermore, diversity was generally higher in herbivores [such as ruminants (i.e., cattle in our study)] than omnivores or carnivores (Reese and Dunn, 2018). Additional data from other sites and individuals may improve our understanding of major drivers of fecal microbiome diversity.

For example, the ANCOM identified a specific ASV belonging to the Fusobacteria phylum and more precisely to the genus *Cetobacterium*, to be associated with wild birds, which may be particularly relevant to the development of a new general avian marker (Supplementary Table 5). Consistent with this observation, Youngblut et al. (2019) also found that OTUs of the genus *Cetobacterium* were associated with animals other than mammals, such as birds. A perspective to this study will be the identification of a qPCR marker from this ASV to complement the general avian MST toolbox.

Indeed, general avian markers have already been developed, and if they are specific (>94%), they often lack significant sensitivity [30–58% for the GFD marker targeting *Helicobacter* in United States and Australia (Green et al., 2012; Ahmed et al., 2016) and 46.5% for the AV4143 marker targeting the *Lactobacillu*s genus in France (*n* = 144; wild bird and poultry samples; Brittany; data not shown)]. However, this dataset confirms that the targeted sequences are present in several avian species. *Helicobacter* spp., the genus targeted by the GFD marker, was mostly found in wild birds (> 20% of the samples from Anatidae, Laridae, and Haematopodidae). The *Lactobacillus* genus targeted by the AV4143 marker was detected in both wild (mainly Brent goose, mallards, and mute swans) and domestic (geese and swans) Anatidae, in poultry (mainly chickens, turkeys, and guinea fowls) and in seagulls, curlews, and cormorants, confirming its status as a general avian source marker.

### Fecal Microbiomes of Wild Waterbirds and MST Markers

In this study, we described the bacterial communities’ structure of a large number of fecal samples (*n* = 425), most of them (*n* = 275) representing the main wild waterbird families (i.e., Anatidae, Laridae, Scolopacidae, Haematopodidae, Phalacrocoracidae, and Hydrobatidae) that are present on coastal areas in Brittany (France) and likely to be a source of pathogenic microbes to bathing waters or shellfish harvesting areas. We compared them to fecal microbiomes from a selection of poultry, livestock animals (cattle and pigs), and influents/effluents of WWTPs (*n* = 150) with the secondary aim of selecting potential MST qPCR markers.

Our bioinformatic analysis used the Nextflow-based SAMBA pipeline^7^, which is based on the approach of ASVs rather than OTUs. The ASV concept is based on sequence variant that provides finer resolution consistent with biological significance (Callahan et al., 2016). In fact, to avoid the ecological limitations of the 97% threshold-based OTU method, we applied ASVs as recommended by Callahan et al. (2016), which is a threshold-free metric of classification. In addition, this workflow performs a distribution-based OTU calling after DADA2, which reduces the bias in identifying false-positive ASVs and thus enables the real diversity of the samples to be described as accurately as possible.

Here, sequencing data were analyzed at the bacterial phylum (Figure 4 and Supplementary Figures 2, 3), family (Supplementary Figures 8, 9), and genus (Figure 3 and Supplementary Figure 4) classification levels. From our results, we stated that fecal bacterial communities’ structure within the 15 wild waterbird species was significantly divergent compared to the other investigated fecal sources, suggesting that DNA sequences specific to wild birds could be identified and allowed the development of markers associated with certain wild avian hosts. Firmicutes, Proteobacteria, and Fusobacteria were the dominant phyla in wild waterbird samples, which corroborates findings from previous studies (Waite and Taylor, 2015; Ohad et al., 2016; Wang et al., 2018; Liao et al., 2019).

The Proteobacteria phylum was ubiquitous, with a predominance in wild waterbirds compared to chicken droppings and cattle and pig feces. Fusobacteria was found predominant in the common shelduck group (*Tadorna tadorna*, Anatidae), which differs from the other bird species. However, this result was different from common shelduck fecal samples analyzed in China where Proteobacteria was found as the dominant phylum (90.6%) (Cao et al., 2020). The Actinobacteria phylum is widely distributed in the environment, including soils, fresh and marine waters, and GI tracts of animals (Janssen, 2006; Barka et al., 2016). In our study, it was the fifth dominant phylum in the whole dataset, while scarce data are available on its distribution within wild birds. One of the aims of this study was to explore fecal bacterial communities of poorly investigated bird species such as storm petrels or European shags. Interestingly, the Hydrobatidae family represented by storm petrels in our dataset was found dominated by Actinobacteria (40.8%), followed by Firmicutes (38.7%) and Proteobacteria (17.3%).

Within the Firmicutes phylum, *C. marimammalium* is the bacterial species targeted by several gull-associated qPCR markers (i.e., Gull2 and Gull4). In our study, it was detected mainly in seagulls (great black-backed, herring, and black-headed gulls, and *Larus* sp. and *Chroicocephalus* spp.) but also in shorebirds (oystercatchers, curlews, and knots) and, in a lesser extent, in species belonging to wild Anatidae (Brent goose, common shelducks, mallards, and mute swans), indicating that this bacterial species may have a larger broad-spectrum presence in GI of wild waterbirds. These results agreed with data from Laviad-Shitrit et al. (2019), where black-headed gulls harbored high abundance of the genus *Catellicoccus* (58.8%), while Gull2 and Gull4 markers were found with high incidence in gulls and shorebirds in the United States (Ryu et al., 2012, 2014). In the same way, *Helicobacter* spp., the genus targeted by the general avian marker GFD (Green et al., 2012), was found mostly in the wild birds of our dataset (>20% of the samples from Anatidae, Laridae, and Haematopodidae).

Rare studies developed avian-associated MST markers from the phylum Bacteroidetes (Kobayashi et al., 2013), even if members of Bacteroidetes have been found in relatively high abundance in fecal microbiomes of few waterbirds like wild geese (Wang et al., 2018). In our study, a low relative abundance (<5%) of this phylum was observed in birds except oystercatchers (15.6%) and mallards (*Anas*, 20.1%), which corroborates data from Grond et al. (2018), which attributed it to dietary differences.

In our study, the ANCOM analysis allowed us to identify several potential new MST markers from wild waterbirds (Supplementary Table 5) including Swan_2 and Oyscab targeting *Romboutsia* (Firmicutes phylum) and *Bacteroides* genera, respectively, which were the most promising ones. While the *Romboutsia* genus was mainly found in wild waterbirds, it was predominant in swans, which was confirmed by the ANCOM analysis. The qPCR assays targeting Swan_2 gave a sensitivity of 75% and a specificity of 90.2%. A broader validation with extensive sample size and geographic sampling locations will improve the obtained data.

### Fecal Microbiomes of Poultry, Livestock Animals, and WW, and MST Markers

Poultry fecal microbiomes were dominated by Firmicutes, Bacteroidetes, Actinobacteria, and Proteobacteria. This is in line with previously published metataxonomic data for the chicken and turkey GI microbiomes (Qu et al., 2008; Yeoman et al., 2012; Oakley et al., 2013; Wei et al., 2013; Choi et al., 2014; Mohd Shaufi et al., 2015; Borda-Molina et al., 2016). However, a higher average relative abundance of Firmicutes was observed in poultry than in wild waterbirds as previously described by Grond et al. (2018). At our best knowledge, no study addressing Firmicutes function in wild birds was available, while in domestic chicken, several studies found a positive relationship between Firmicutes abundance and mass gain and immune function, suggesting similar roles of Firmicutes between mammals and birds (Liao et al., 2015; Zhang et al., 2015).

Only one bacterial poultry marker has been developed to our knowledge: the AV43 marker developed by Ohad et al. (2016) from high-throughput sequencing data that was found to be specific in their study (91% sensitivity). However, the taxonomic affiliation only to phylum Firmicutes did not allow searching for this marker or the targeted DNA sequence in our dataset.

Within Actinobacteria, *Brevibacterium*, the genus targeted for the poultry litter qPCR marker LA35 (Weidhaas et al., 2010), was ubiquitous in poultry litter samples (in chicken, from 1 to 18% of reads per sample, and in turkey, from 1 to 4%), whereas it was only present in 10 out of the 17 chicken droppings (from 0.1 to 2.2%), and absent in the turkey droppings, confirming the specificity of the *Brevibacterium* genus to poultry litter samples.

Most MST effort was made on human and livestock animals where bacteria within the phylum Bacteroidetes, and in particular the bacterial order *Bacteroidales*, were the major focus (Bernhard and Field, 2000; Mieszkin et al., 2009; Boehm et al., 2013; Reischer et al., 2013). In our study, this phylum was found in all cattle samples, in more than 70% of the pig samples, and wastewater samples.

In our study, the ANCOM analysis confirmed the status of the well-known human (HF183), pig (Pig2Bac), and cattle (Rum2Bac) MST markers. Furthermore, this ANCOM analysis showed that ASVs belonging to the Ruminococcaceae family (Firmicutes) could be an interesting target for new MST markers for cattle, in agreement with Youngblut et al. (2019) who find genera of this family exclusively in mammals.

### Genera With Bacterial Pathogens and MST Markers

Although wild bird droppings appear to harbor fewer pathogenic bacteria and in a weaker frequency than the wastes from poultry, livestock animals, and the influents/effluents of WWTPs, their incidence should not be neglected (Benskin et al., 2009). Studying wild waterbird fecal microbiomes could be useful to have an overview of potential bacterial pathogens harbored by these hosts. This method makes it possible to screen a larger number of pathogens than by using culture-based methods targeting independently pathogens. We found that the composition and the diversity of potential pathogen genera vary significantly between hosts, as observed by Fu et al. (2020). Thus, we observed frequent presence of certain genera that include pathogenic species such as *Clostridium sensu stricto 1*, *Campylobacter*, *Fusobacterium*, and *Helicobacter* in the bird droppings. Such genera were also identified within bacterial communities from several waterbird species including great cormorants, little egrets, black-crowned night herons, and black-headed gulls (Laviad-Shitrit et al., 2019).

As reported in several studies using either 16S rRNA gene sequencing (high-throughput or cloning; Grond et al., 2014; Ryu et al., 2014; Laviad-Shitrit et al., 2019) or by culture-based methods (Waldenstrom et al., 2007; More et al., 2017; Vogt et al., 2018), *Campylobacte*r spp. were found to be frequent in bird fecal microbiomes analyzed in this study. Thus, using a culture-based method, we isolated several *Campylobacte*r spp. strains in half of the wild waterbird droppings samples including Brent goose, common shelducks, mute swans, oystercatchers, curlews, knots, great black-backed, and black-headed gulls (data not shown). Even if the high-throughput sequencing method allows the detection of members of this genus, it rarely allows the taxonomic assignation at the species level (i.e., only *Campylobacter canadensis* identified here). Furthermore, the presence of ASVs that belong to members of genera including potentially human pathogens does not necessarily mean that these ASVs belong to pathogenic species, as suggested by Laviad-Shitrit et al. (2019). Thus, the culture-based isolation of the *Campylobacter* spp. is needed to define the species pathogenic level and evaluate their pathogenicity by *in silico* (screening antibiotic resistance and virulence genes) or *in vitro* (insect or animal models) assays. *Clostridium sensu stricto 1*, another genus harboring human pathogens, was mainly found in wild Anatidae, Phalocrocoracidae, and Scolopacidae (>50% of the samples). This is consistent with previous studies where this genus was commonly found in migratory wild waterbirds (hooded crane and greater white-fronted goose) in China (Xiang et al., 2019) and Israel (Laviad-Shitrit et al., 2019). Members of the genus *Fusobacterium* were present in more than half of the droppings of Brent geese, mallards, oystercatchers, black-headed gulls, and great cormorants, while its relative abundance was very low in black-headed gull samples (<1%). These results are consistent with the results obtained in the study of Laviad-Shitrit et al. (2019) in which *Fusobacterium* was found ubiquitous in the studied bird species with high prevalence (19.8–32.4%) except for the black-headed gulls (0.01%) (Grond et al., 2019). In contrast, other genera were minor, including *Mycoplasma* that was detected in breeding ducks and mute swans. While few species are known as pathogens in birds, *M. gallisepticum* was detected in numerous wild bird species (Sawicka et al., 2020).

The detection and enumeration of all pathogenic microorganisms potentially present is technically impossible. Thus, for routine water quality monitoring, FIB are usually enumerated to evaluate the level of microbial contamination from a fecal source (Ashbolt et al., 2001). However, it is well known that they have not the same distribution as pathogens (Stewart et al., 2008), so it is necessary to develop new MST markers of yet undefined sources. Here, we identified key correlations, i.e., strong correlation between *Catellicoccus*, which harbors bird markers, and *Fusobacterium*, which is widely detected in birds and known to harbor pathogens. While most studies on the relationships between MST markers and pathogens were achieved using qPCR assays for environmental sinks (Vadde et al., 2019), their status for intra-fecal sources using microbiome data in our study is less clear and needs to be further investigated.

In summary, several hypotheses could be stated to explain microbiome diversity and pathogen distribution within the hosts, such as the bird species diversity, and their variable life-history, such as migratory behavior, diet, and physiology, all of which may impact gut microbiota (Grond et al., 2018). While our dataset included 15 different wild waterbird species, the main limitation was the difficulty in collecting similar numbers of samples per bird species. Simultaneously, culture-based methods were applied for enriching *Campylobacter* members from the collected fecal samples, which helps in evaluating the prevalence of pathogenic species from this genus and their pathogenicity. Future work will include metabarcoding data from other sites, including waters from bathing and shellfish areas that may help in evaluating the coalescence of bacterial communities using the SourceTracker Bayesian approach and mitigation fecal pollution from yet unknown sources.

## Conclusion

This study provides a comprehensive snapshot of the gut bacterial diversity in a selection of wild waterbirds compared to poultry, cattle, pigs, and wastewater samples, using the NGS-based 16S rRNA gene amplicon method. Although fecal markers have been identified and developed for source tracking applications in many countries, the microorganism communities in GI tracts can be dissimilar for each host species in different geographical areas, which may be due to climate, food, behavior, antibiotics, and other region-specific factors. This potential variation can lead to variability in the performance of fecal markers among regions. High abundances of phyla including Firmicutes, Proteobacteria, and Fusobacterium (especially in wild waterbirds, for this latest phylum), which encompass several genera including potential pathogens, were detected. Moreover, alpha diversity indices and NMDS plots indicated more similarity in the gut microbiota within waterbirds, while they were highly different from the other sources. Comparison to the non-bird sources led us to select several unique and host-associated ASVs related to one or multiple groups of wild waterbirds to be used as MST candidates including the Swan_2 marker. Further analyses are required to improve our findings, while other strategies (i.e., SourceTracker) can be used to help in developing prediction models.

## Data Availability Statement

The datasets presented in this study can be found in online repositories. The names of the repository/repositories and accession number(s) can be found in the article/Supplementary Material.

## Author Contributions

MG and AB coordinated and designed the experiments. AB, BC, JC, and MG participated in the collection of fecal samples. CN, LQ, and AC developed the SAMBA workflow. AB, MG, LQ, and CN performed the bioinformatic data analysis. AB, EQ, LD, and MG performed the experiments and contributed to the analysis of the datasets. AB and MG prepared the manuscript with contributions from all co-authors. All the authors read and approved the final version of the manuscript.

## Conflict of Interest

The authors declare that the research was conducted in the absence of any commercial or financial relationships that could be construed as a potential conflict of interest.

## Footnotes

1 avibase.bsc-eoc.org

2 https://github.com/ifremer-bioinformatics/samba

3 https://blast.ncbi.nlm.nih.gov/Blast.cgi

4 https://primer3.ut.ee/

5 https://health.canada.ca/en/epathogen

6 https://doi.org/10.12770/ddce4bf7-2b71-4929-aa20-50a8fd0e706a

7 https://github.com/ifremer-bioinformatics/samba
