# Supplementary material for: Comparative Analysis of Fecal Microbiomes From Wild Waterbirds to Poultry, Cattle, Pigs, and Wastewater Treatment Plants for a Microbial Source Tracking Approach
Source: Front Microbiol. 2021 Jul 14;12:697553. doi: 10.3389/fmicb.2021.697553 (PMC8317174; doi:10.3389/fmicb.2021.697553)
Supplement: Supplementary file 1 [file Data_Sheet_1.PDF]

Supplementary data

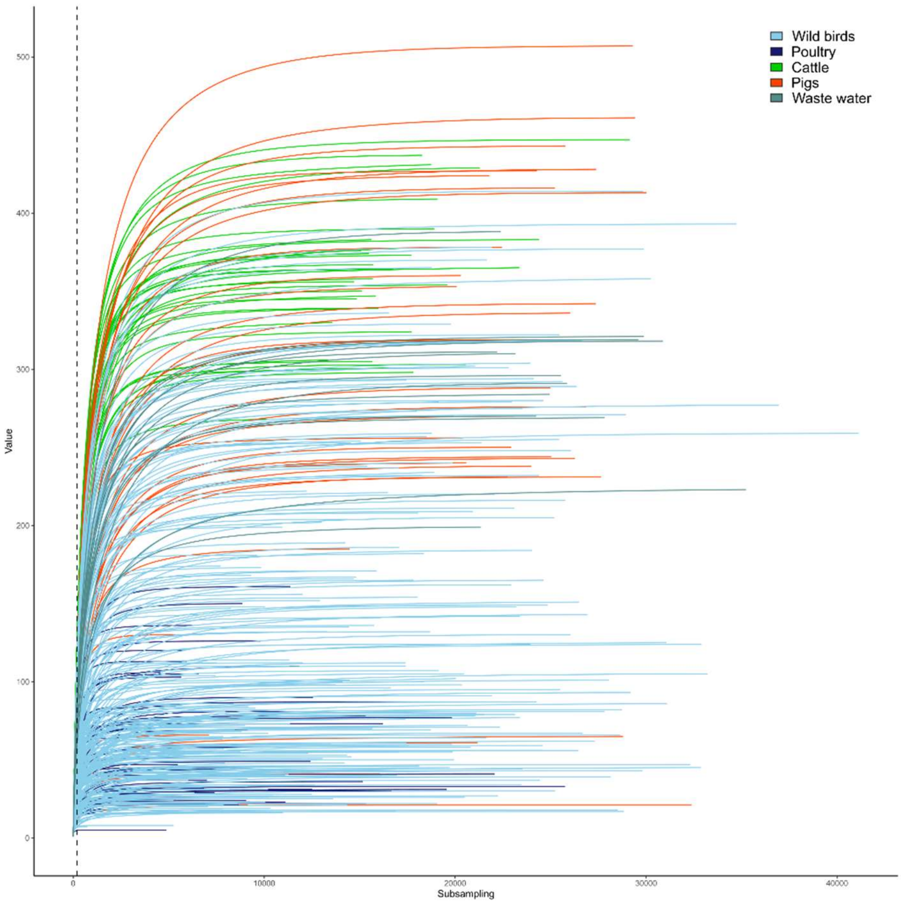

Supplementary Figure S1**.** Rarefaction curves showing the relation between the number of V3-V4 16S rRNA gene reads analyzed and ASV numbers per group.

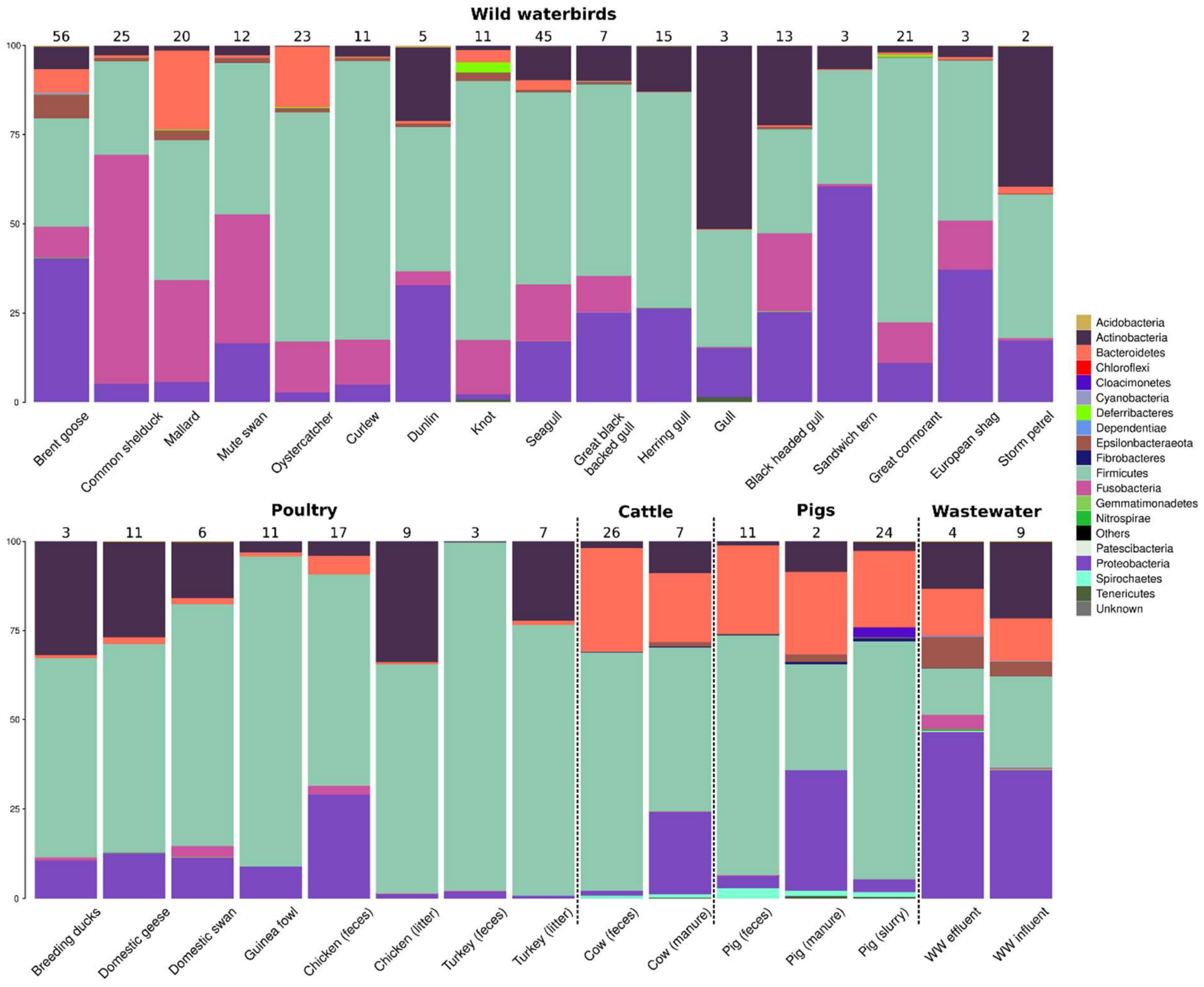

Supplementary Figure S2. Stacked bar charts showing the mean relative abundances of the eighteen most predominant bacterial phyla within the five groups of fecal samples. Percent sequence abundances given as the number of reads matching a given bacterial phyla per total reads for that sample.

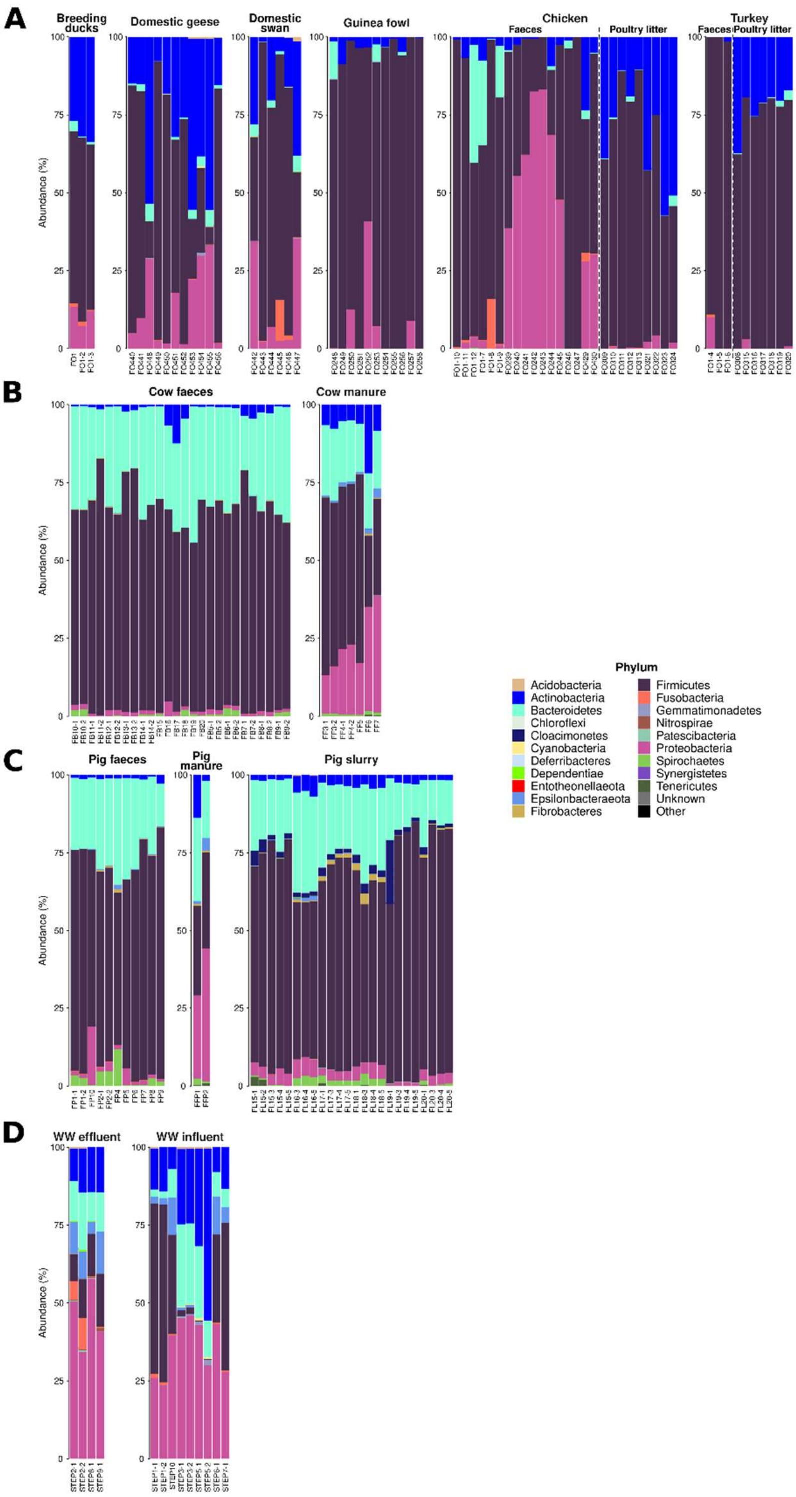

Supplementary Figure S3. Distribution of predominant bacterial phyla in poultry, cattle, pigs and wastewater samples according to relative abundances obtained by the gene encoding 16S rRNA. Stacked bar plots represent the sequence abundances of the twenty most abundant phylum-level taxa identified in the fecal samples. Percent sequence abundances given as the number of reads matching a given bacterial phyla per total reads for that sample.

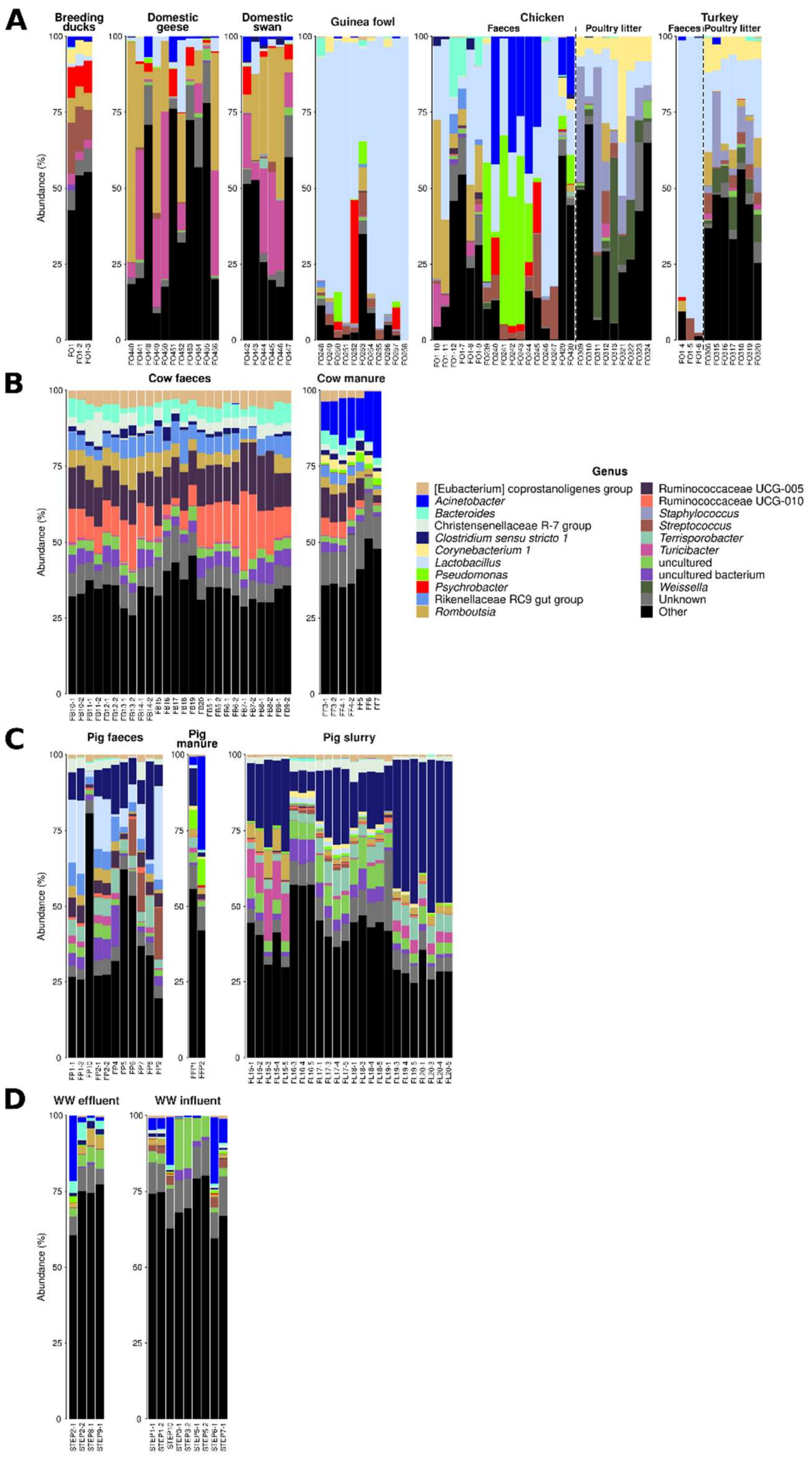

Supplementary Figure S4. Distribution of predominant bacterial genera in poultry, cattle, pigs and wastewater samples according to relative abundance obtained by the gene encoding 16S rRNA. Stacked bar plots represent the sequence abundances of the twenty most abundant genus-level taxa identified in the fecal samples. Percent sequence abundances given as the number of reads matching a given bacterial genus per total reads for that sample.

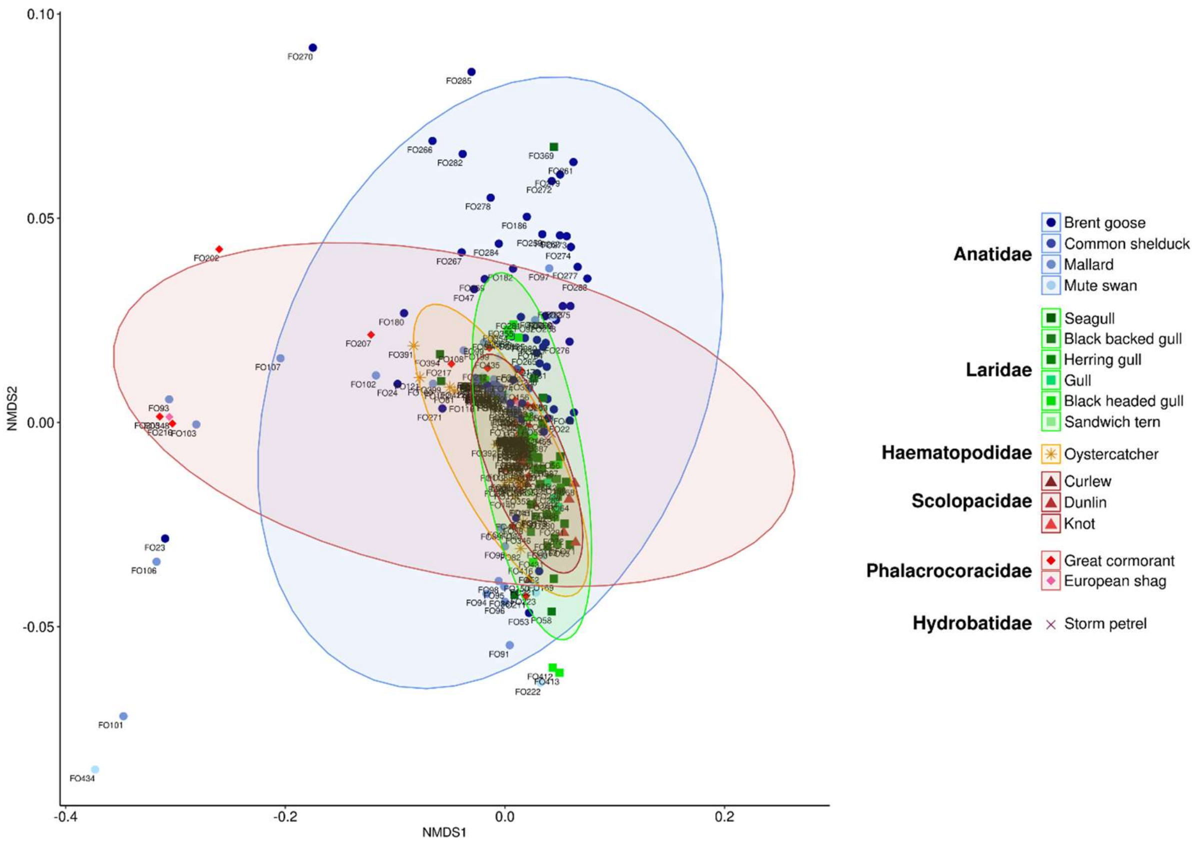

Supplementary Figure S5. Non-metric multidimensional scaling (NMDS) plots based weighted Unifrac distance metric (stress=0.126) in relation to wild waterbirds groups. Colors represent host classes. Ellipses represent 95% confidence intervals of centroids of each point.

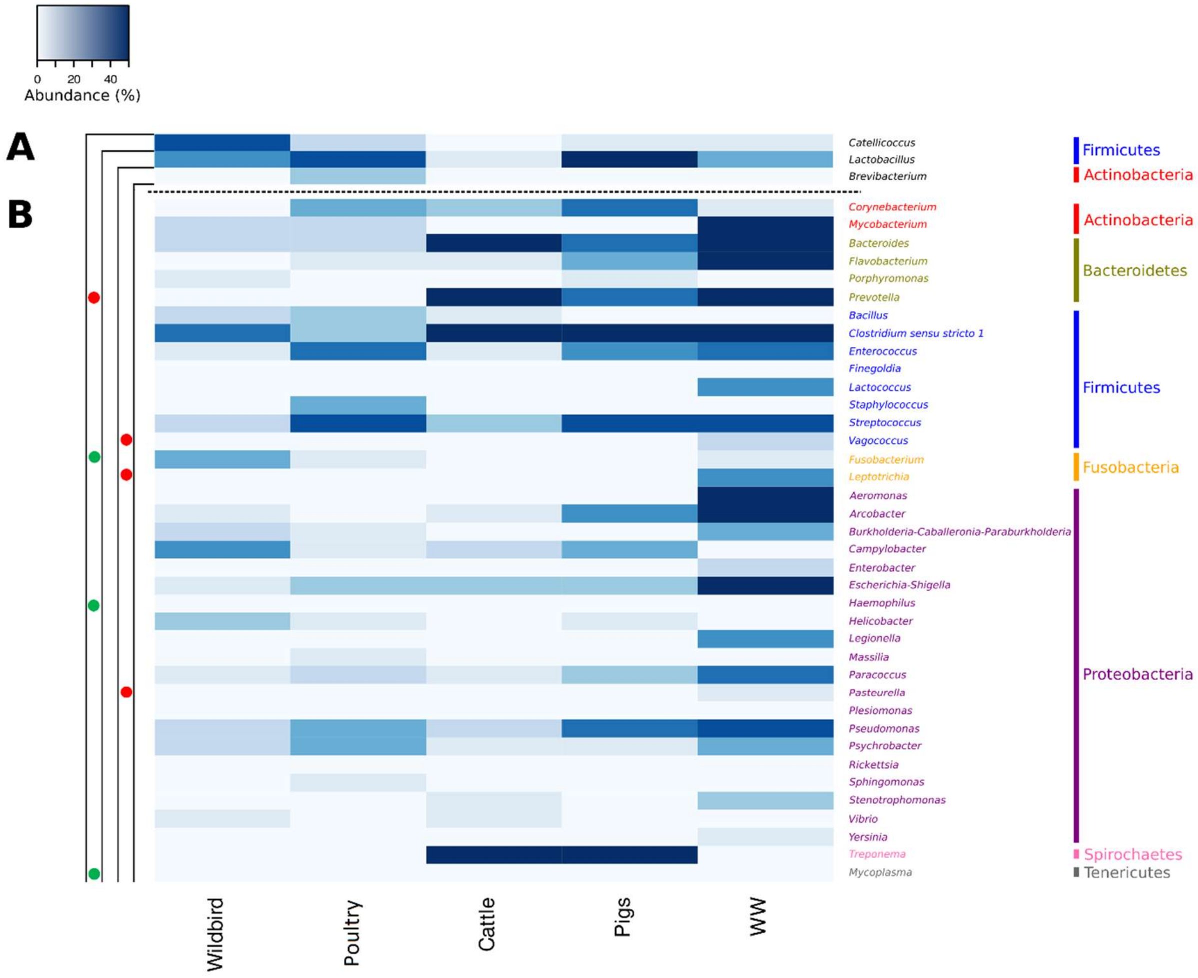

Supplementary Figure S6. Heatmaps based on the number of reads of a selection of (**A**) three genera harboring known MST markers and (**B**) thirty-seven bacterial genera harboring potential pathogens, and derived from the whole dataset at the groups levels according to the source (WW: wastewater).

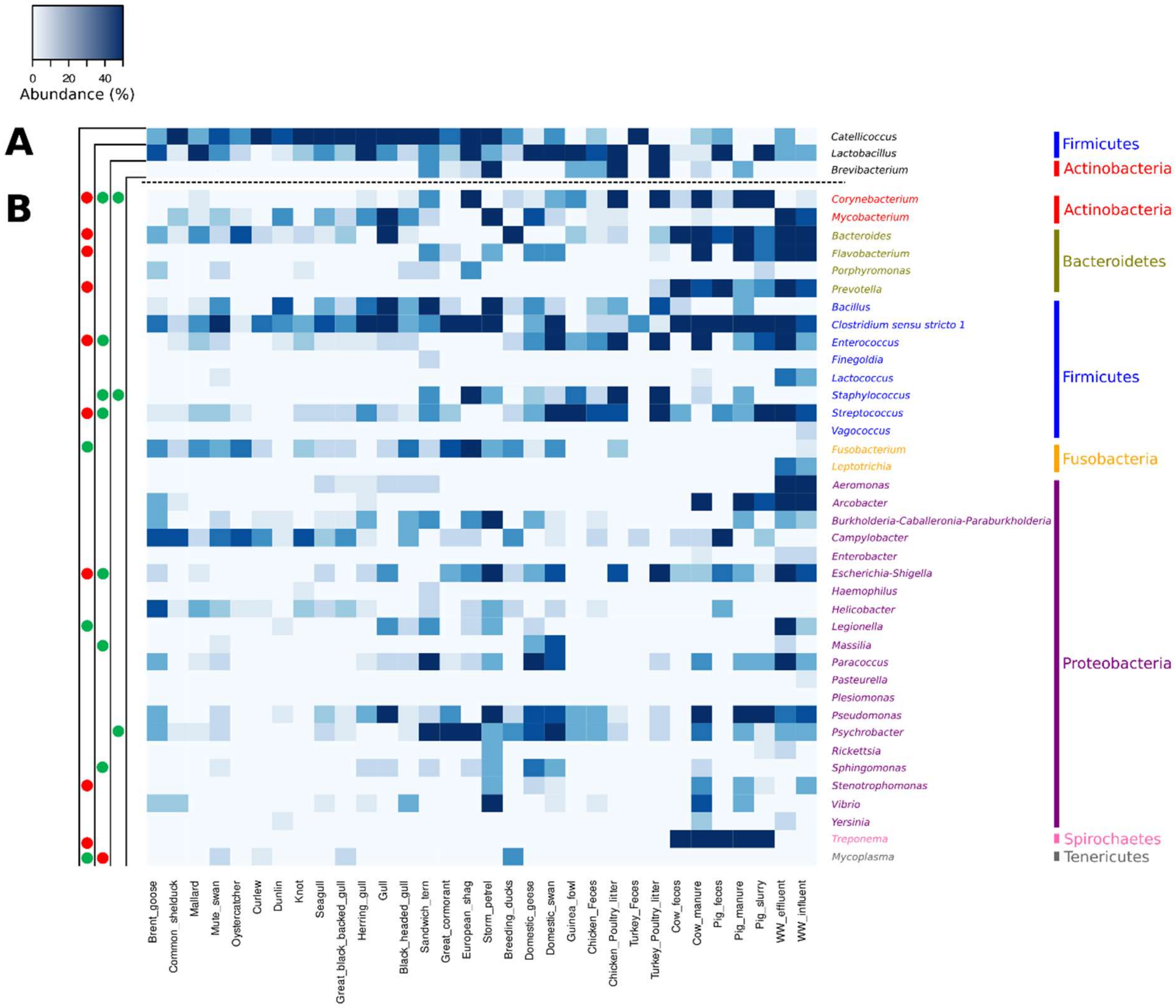

Supplementary Figure S7. Heatmaps based on the number of reads of a selection of three genera harboring known MST markers (**A**) and thirty-seven bacterial genera harboring potential pathogens (**B**), and derived from the whole dataset at the detailed sources level.

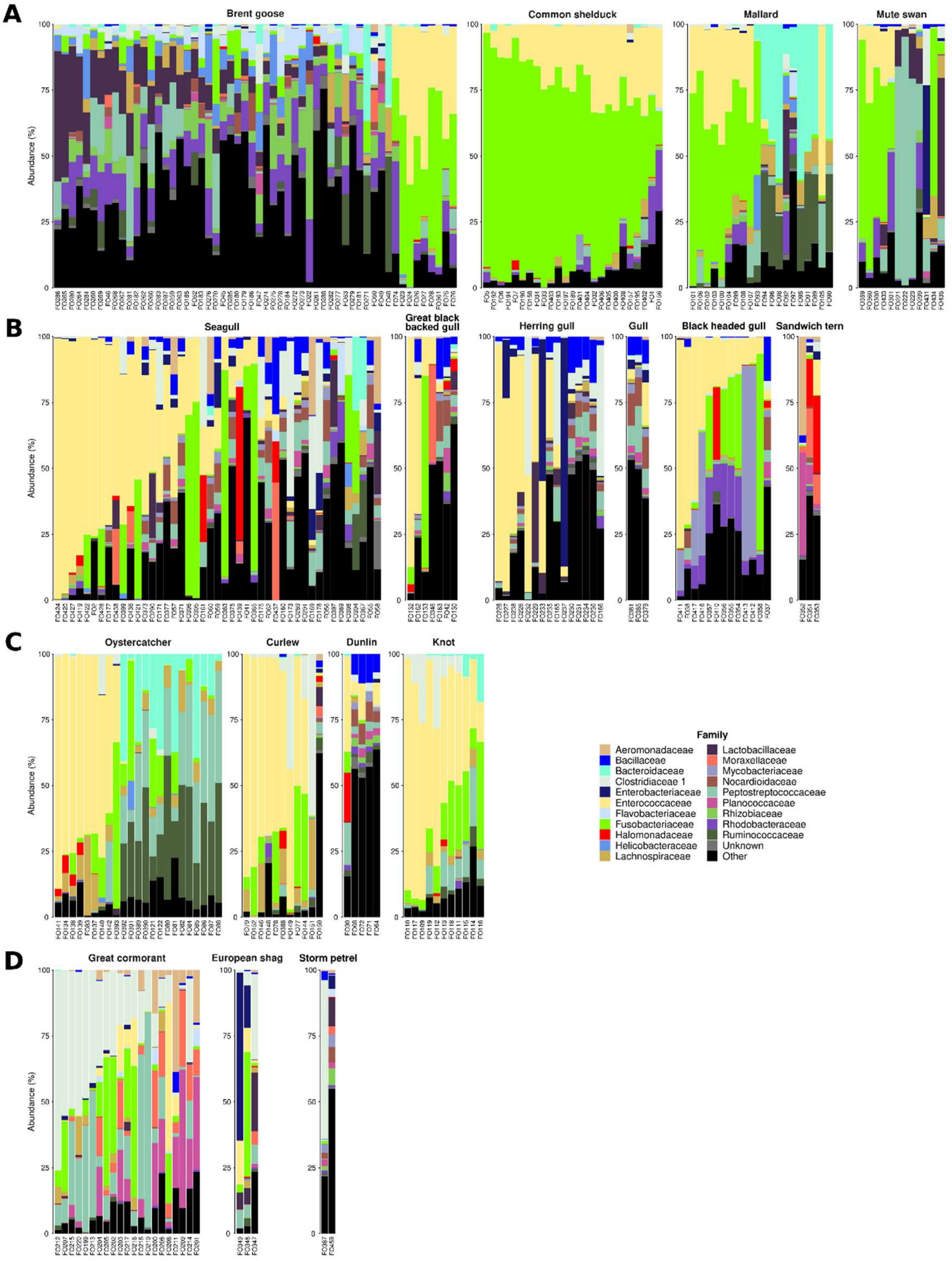

Supplementary Figure S8. Distribution of predominant bacterial families in wild waterbirds according to relative abundance obtained by the gene encoding 16S rRNA. Stacked bar plots represent the sequence abundances of the 18 most abundant family-level taxa identified in the fecal samples. Percent sequence abundances given as the number of reads matching a given bacterial family per total reads for that sample. Bacterial community compositions were grouped by wild waterbirds families: (**A**) wild Anatidae, (**B**) Laridae, (**C**) Haematopodidae and

Scolopacidae (wader birds) and (**D**) Phalacrocoracidae and Hydrobatidae (cormorants and storm petrels, respectively).

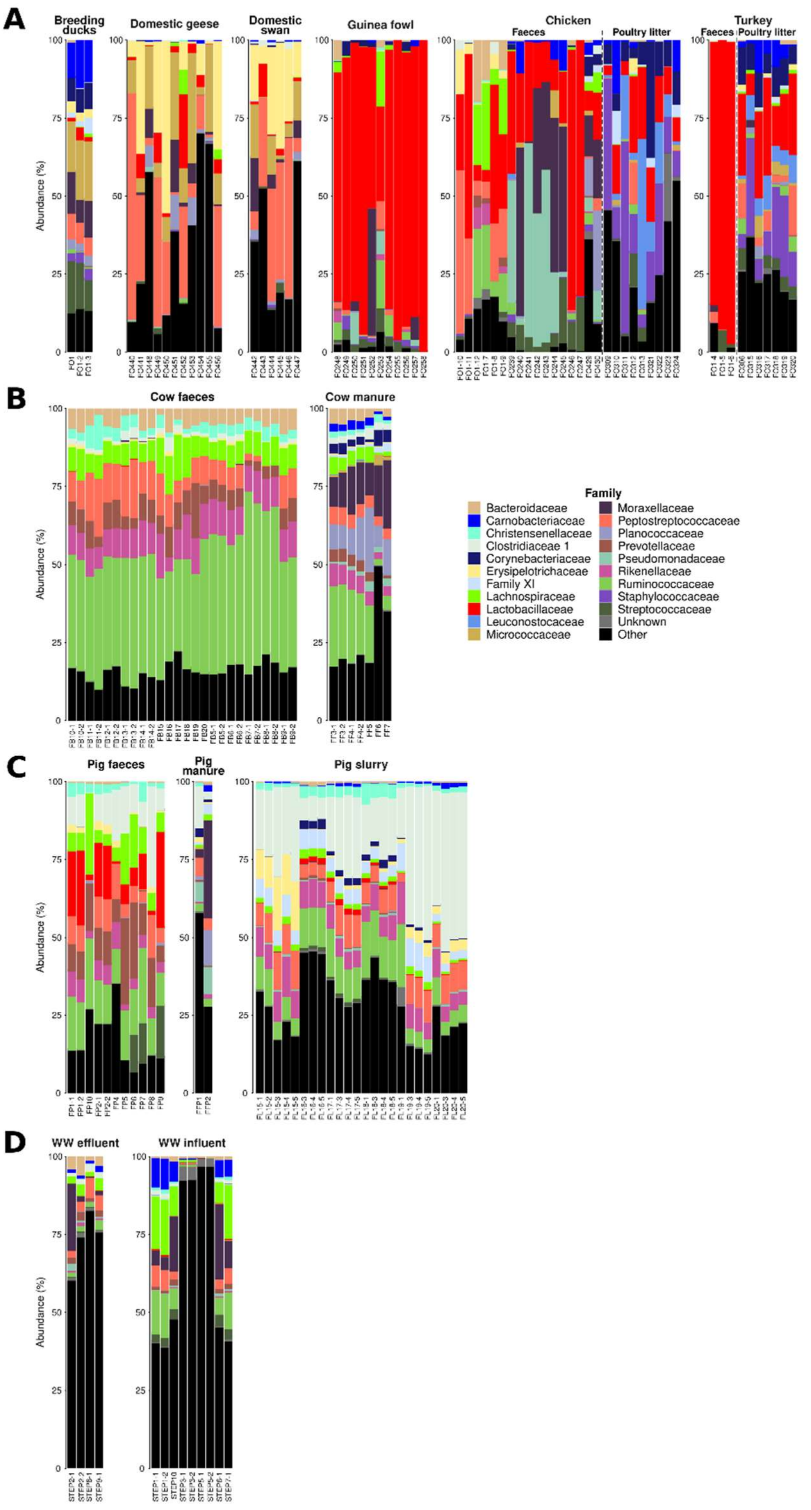

Supplementary Figure S9. Distribution of predominant bacterial families in poultry (**A**), cattle (**B**), pigs (**C**) and wastewater (**D**) samples according to relative abundance. Stacked bar plots represent the sequence abundances of the 18 most abundant family-level taxa identified in the fecal samples. Percent sequence abundances given as the number of reads matching a given bacterial family per total reads for that sample.
