# Supplementary material for: Comparative Analysis of Fecal Microbiomes From Wild Waterbirds to Poultry, Cattle, Pigs, and Wastewater Treatment Plants for a Microbial Source Tracking Approach
Source: Front Microbiol. 2021 Jul 14;12:697553. doi: 10.3389/fmicb.2021.697553 (PMC8317174; doi:10.3389/fmicb.2021.697553)
Supplement: Supplementary file 2 [file Data_Sheet_2.PDF]

Supplementary Table S1**.** Description of the fecal samples collection.

Families, genera and species	Name	Diet	Diet (detailed)	Wild/domestic	No. of birds/animals tested	Types of samples	Status in Brittany
**Laridae**					**86**		
**Larinae** (sub-family)					**83**		
Chroicocephalus	Gull	Omnivore	Opportunistic omnivorous feeder	Wild	3	Feces	
Chroicocephalus ridibundus	Black headed gull	Omnivore	Opportunistic omnivorous feeder	Wild	13	Feces	Breeding / Migration / Winter
Larus	Seagull	Omnivore	Opportunistic omnivorous feeder	Wild	45	Feces	
Larus argentatus	Herring gull	Omnivore	Opportunistic omnivorous feeder	Wild	15	Feces	Breeding / Migration / Winter
Larus marinus	Great black backed gull	Omnivore	Opportunistic omnivorous feeder	Wild	7	Feces	Breeding / Sedentary
**Sternidae** (sub-family)					**3**		
Thalasseus sandvicensis	Sandwich tern	Carnivore	Fish-eating species and invertebrate feeder	Wild	3	Feces	Breeding / Migration / Winter
**Phalacrocoracidae**					**24**		
Phalacrocorax aristotelis	European shag	Carnivore	fish-eating species	Wild	3	Feces	Breeding / Sedentary
Phalacrocorax carbo	Great cormorant	Carnivore	fish-eating species	Wild	21	Feces	Breeding / Migration / Winter
**Scolopacidae**					**27**		
Calidris alpina	Dunlin	Carnivore	shoreline-foraging invertebrate feeder	Wild	5	Feces	Migration / Winter
Calidris canutus	Red knot	Carnivore	shoreline-foraging invertebrate feeder	Wild	11	Feces	Migration / Winter
Numenius arquata	Curlew	Carnivore	shoreline-foraging invertebrate feeder	Wild	11	Feces	Breeding / Migration / Winter
**Haematopodidae**					**23**		
Haematopus ostralegus	Oystercatcher	Carnivore	Aquatic invertebrate feeder	Wild	23	Feces	Breeding / Migration / Winter
**Anatidae**					**133**		
Anas platyrhynchos	Mallard	Omnivore	Aquatic invertebrate and seed feeder	Wild	20		Breeding / Migration / Winter
Branta bernicla	Brent goose	Herbivore	Aquatic plant-eating species	Wild	56	Feces	Migration / Winter
Cygnus olor	Mute swan	Herbivore	Aquatic plant-eating species	Wild	12	Feces	Breeding / Migration / Winter
Tadorna tadorna	Common shelduck	Omnivore	Algae and aquatic invertebrate feeder	Wild	25	Feces	
Anas	Breeding duck	Omnivore	Seed feeder	Domestic	3	Feces	
Anser anser	Greylag goose	Herbivore	Aquatic plant-eating species	Domestic	1	Feces	
Anser brachyrhynchus	Pink footed goose	Herbivore	Aquatic plant-eating species	Domestic	1	Feces	

Supplementary Table S1. *Continued.*

Families, genera and species	Name	Diet	Diet (detailed)	Wild/domestic	No. of birds/animals tested	Types of samples	Status in Brittany
Anser erythropus	Lesser white front goose	Herbivore	Aquatic plant-eating species	Domestic	1	Feces	
Anser indicus	Bar headed goose	Herbivore	Aquatic plant-eating species	Domestic	1	Feces	
Branta canadensis	Canada goose	Herbivore	Aquatic plant-eating species	Domestic	1	Feces	
Branta leucopsis	Barnacle goose	Herbivore	Aquatic plant-eating species	Domestic	1	Feces	
Branta sandvicensis	Hawaiian goose	Herbivore	Aquatic plant-eating species	Domestic	1	Feces	
Chen caerulescens	Snow goose	Herbivore	Aquatic plant-eating species	Domestic	1	Feces	
Chen rossii	Moss goose	Herbivore	Aquatic plant-eating species	Domestic	1	Feces	
Cyanochen cyanoptera	Blue winged goose	Herbivore	Aquatic plant-eating species	Domestic	1	Feces	
Cygnus atratus	Black swan	Herbivore	Aquatic plant-eating species	Domestic	2	Feces	
Cygnus immutabilis	Polish mute swan	Herbivore	Aquatic plant-eating species	Domestic	2	Feces	
Cygnus melancoryphus	Black necked swan	Herbivore	Aquatic plant-eating species	Domestic	2	Feces	
Neochen jubata	Orinoco goose	Herbivore	Aquatic plant-eating species	Domestic	1	Feces	
**Hydrobatidae**					**2**		
Hydrobates pelagicus	Storm petrels	Carnivore	Planktonophagous and fish-eating species	Wild	2	Feces	Breeding / Migration
**Numididae**					**11**		
Numida meleagris	Guinea fowls	Omnivore	Seed feeders	Domestic	11	Feces	
**Phasianidae**					**36**		
Gallus	Chicken/hens	Omnivore	Seed feeders	Domestic	17	Feces	
					9	Poultry Litter	
Meleagris	Turkey	Omnivore	Seed feeders	Domestic	3	Feces	
					7	Poultry Litter	
**Bovidae**					**33**		
Bos taurus	Cattle	Herbivore	Plant-eating	Domestic	**26**	Feces	
					7	Solid manure	
**Suidae**					**37**		
Sus scrofa domesticus	Pigs	Omnivore	omnivorous	Domestic	**11**	Feces	

Supplementary Table S1. *Continued*.

Families, genera and species	Name	Diet	Wild/domestic	No. of birds/animals tested	Types of samples	Status in Brittany
				**2**	Solid manure	
				24	Liquid manure	
**Wastewaters (WW)**				**13**		
WW	Influents/effluents	NA		**9**	Influents	
				4	Effluents	
**Total**				**425**		

NA. not applicable.

Supplementary Table S2**.** Metadata on the collected fecal samples from wild birds.

Bird species	Scientific nomenclature	# of fecal samples	Geographic distribution	#	Date	#
Mallard	*Anas platyrhynchos*	20	Côtes-d'Armor	20	08/02/2017	20
Brent goose	*Branta bernicla*	56	Côtes-d'Armor	19	01/12/2016	4
			Finistère	7	12/12/2016	3
			Gironde	30	30/01/2017	11
					31/01/2017	3
					02/02/2017	10
					15/02/2017	16
					27/03/2017	8
					19/06/2017	1
Dunlin	*Calidris alpina*	5	Côtes-d'Armor	4	30/01/2017	5
			Finistère	1		
Knot	*Calidris canutus*	11	Côtes-d'Armor	11	14/02/2017	11
Gull	*Chroicocephalus*	3	Côtes-d'Armor	3	10/07/2017	3
Black-headed gull	*Chroicocephalus ridibundus*	13	Côtes-d'Armor	6	30/01/2017	2
			Finistère	7	19/06/2017	5
					26/09/2017	6
Mute swan	*Cygnus olor*	12	Charente-Maritime	7	22/02/2017	3
			Finistère	5	27/04/2017	3
					19/06/2017	2
					10/10/2017	4

Supplementary Table S2. ***Continued.***

**Bird species**	**Scientific nomenclature**	**# of fecal samples**	**Geographic distribution**	**#**	**Date**	**#**
Oystercatcher	*Haematopus ostralegus*	23	Côtes-d'Armor	23	31/01/2017	9
					14/02/2017	2
					20/03/2017	7
					26/09/2017	5
Storm petrel	*Hydrobates pelagicus*	2	Finistère	2	23/08/2017	2
Seagull	*Larus*	45	Charente-Maritime	4	29/07/2016	1
			Côtes-d'Armor	31	30/01/2017	8
			Finistère	9	27/03/2017	8
			Hérault	1	27/04/2017	3
					19/06/2017	3
					10/07/2017	5
					26/09/2017	13
					10/10/2017	4
Herring gull	*Larus argentatus*	15	Finistère	15	27/03/2017	2
					18/04/2017	13
Black-backed gull	*Larus marinus*	7	Côtes-d'Armor	3	30/01/2017	1
			Finistère	4	20/03/2017	3
					27/03/2017	2
					24/05/2017	1
Curlew	*Numenius arquata*	11	Côtes-d'Armor	11	31/01/2017	3
					20/03/2017	7
					26/09/2017	1

Supplementary Table S2. ***Continued.***

**Bird species**	**Scientific nomenclature**	**# of fecal samples**	**Geographic distribution**	**#**	**Date**	**#**
European shag	*Phalacrocorax aristotelis*	3	Finistère	3	18/05/2017	3
Great cormorant	*Phalacrocorax carbo*	21	Charente-Maritime	2	22/02/2017	2
			Finistère	19	28/03/2017	10
					29/03/2017	9
Common shelduck	*Tadorna tadorna*	25	Côtes-d'Armor	15	12/12/2016	4
			Finistère	10	30/01/2017	3
					27/03/2017	10
					26/09/2017	8
Sandwich tern	*Thalasseus sandvicensis*	3	Finistère	3	31/05/2017	3

Supplementary Table S4**.** Total numbers of species and the corresponding pathogenic within the selected 37 genera. This list was validated according to the Canadian ePATHogen risk group database (https://health.canada.ca/en/epathogen, accessed on February 17th 2021).

Phyla/genus	Total species number	Pathogenic species number[a]
**Actinobacteria**		
Corynebacterium	152	32
Mycobacterium	211	90
**Bacteroidetes**		
Bacteroides	102	21
Flavobacterium	274	3
Porphyromonas	20	6
Prevotella	58	18
**Firmicutes**		
Bacillus	440	10
Clostridium	247	38
Enterococcus	65	10
Finegoldia	1	1
Lactococcus	21	2
Staphylococcus	64	26
Streptococcus	138	38
Vagococcus	16	2
**Fusobacteria**		
Fusobacterium	22	17
Leptotrichia	7	3
**Proteobacteria**		
Aeromonas	38	12
Arcobacter	30	4
Burkholderia	178	14
Campylobacter	41	27
Enterobacter	49	10
Escherichia-Shigella	8-6	5-6
Haemophilus	25	10
Helicobacter	50	9
Legionella	64	39
Massilia	55	1
Pasteurella	25	8
Plesiomonas	1	1
Pseudomonas	179	7
Psychrobacter	43	3
Rickettsia	74	23
Sphingomonas	169	5
Stenotrophomonas	19	1
Vibrio	155	14
Yersinia	27	12
**Spirochaetes**		
Treponema	39	17
**Tenericutes**		
Mycoplasma	155	14

[a]Corresponding to pathogens classified as level 2 or above.

Supplementary Table S5. Selected ASVs as potential MST markers with their respective taxonomic allocations, ANCOM scores and GenBank accession numbers.

Selected ASVs	ANCOM score (W)	Family	Genus	Species	GenBank accession
**Species level**					
Mute swan (Swan_2)	7293	Peptostreptococcaceae	*Romboutsia*	unclassified	MZ021257
Oystercatcher (Oyscab)	7339	Bacteroidaceae	*Bacteroides*	unclassified	MZ021258
Mallard	4067	Bacteroidaceae	*Bacteroides*	unclassified	MZ021259
Great cormorant	7339	Peptostreptococcaceae	*Paeniclostridium*	*sordellii*	MZ021260
Storm petrels	7317	Dermacoccaceae	*Flexivirga*	unclassified	MZ021261
Guinea fowl	7339	Lactobacillaceae	*Lactobacillus*	unclassified	MZ021262
**Group level**					
Wild birds	7345	Fusobacteriaceae	*Cetobacterium*	unclassified	MZ021263
Wild birds	7344	Enterococcaceae	*Catellicoccus*	unclassified	MZ021264
Birds	7344	Fusobacteriaceae	Lactobacillus	unclassified	MZ021265
Poultry	7346	Lactobacillaceae	*Lactobacillus*	*salivarius*	MZ021266
Cattle	7348	Ruminococcaceae	UCG-005	unclassified	MZ021267
Cattle	7343	Bacteroidaceae	*Bacteroides*	unclassified	MZ021268
Pig	7348	Erysipelotrichaceae	*Turicibacter*	unclassified	MZ021269
Pig	7344	Rikenellaceae	DMER64	unclassified	MZ021270
Wastewater	7346	Arcobacteraceae	*Arcobacter*	unclassified	MZ021271
Wastewater	7345	Moraxellaceae	*Acinetobacter*	unclassified	MZ021272

Supplementary Table S6**.** Relative proportion of the 31 bacterial phyla (mean ± standard deviation; greater than 0.5%) inferred from the V3-V4 16S rRNA gene dataset over the investigated fecal samples.

Phylum	Wildbirds	Poultry	Cattle	Pigs	WasteWater
**Firmicutes**	47.45±26.83	67.92±25.49	62.81±10.49	64.69±12.96	21.23±20.77
**Proteobacteria**	19.13±20.56	13.26±20.60	4.90±8.01	5.18±8.02	39.44±10.19
**Fusobacteria**	17.03±25.17	0.63±2.52	+	+	1.57±3.00
**Bacteroidetes**	5.02±11.59	2.37±6.04	27.96±7.16	22.56±6.33	12.92±8.25
**Actinobacteria**	8.50±13.92	15.70±16.23	2.96±4.44	2.34±2.38	18.54±13.03
**Epsilonbacteraeota**	2.25±5.49	+	+	+	5.47±5.07
**Tenericutes**	+	+	+	+	nd
**Deferribacteres**	+	+	nd	+	nd
**Cyanobacteria**	+	+	nd	+	+
**Acidobacteria**	+	+	nd	+	+
**Gemmatimonadetes**	+	+	+	+	+
**Fibrobacteres**	+	+	+	0.60±0.76	+
**Dependentiae**	+	+	+	+	+
**Spirochaetes**	+	+	0.83±0.74	1.94±2.20	+
**Chloroflexi**	+	+	+	+	nd
**Nitrospirae**	+	+	nd	nd	+
**Patescibacteria**	+	nd	nd	+	+
**Halanaerobiaeota**	+	nd	nd	nd	nd
**Latescibacteria**	+	nd	nd	nd	+
**Chlamydiae**	+	nd	nd	nd	+
**Calditrichaeota**	+	nd	nd	nd	nd
**Planctomycetes**	+	nd	nd	nd	+
**Schekmanbacteria**	+	nd	nd	nd	nd
**Cloacimonetes**	+	nd	+	1.79±3.39	+
**Entotheonellaeota**	+	+	nd	nd	nd
**MAT-CR-M4-B07**	+	nd	nd	nd	nd
**Kiritimatiellaeota**	+	nd	+	+	nd
**Elusimicrobia**	nd	nd	nd	+	+
**Margulisbacteria**	nd	nd	nd	+	nd
**Marinimicrobia (SAR406 clade)**	nd	nd	nd	+	nd
**Synergistetes**	nd	nd	nd	+	nd

+: relative abundance < 0.5%; nd: not detected.
